# Intracellular *Plasmodium* aquaporin 2 is important for sporozoite production in the mosquito vector and malaria transmission

**DOI:** 10.1073/pnas.2304339120

**Published:** 2023-10-26

**Authors:** Alexander J. Bailey, Chiamaka Valerie Ukegbu, Maria Giorgalli, Tanguy Rene Balthazar Besson, George K. Christophides, Dina Vlachou

**Affiliations:** ^a^Department of Life Sciences, Imperial College London, London SW7 2AZ, United Kingdom

**Keywords:** malaria parasite, mosquito vector, aquaporin, Plasmodium ookinete, oocyst development

## Abstract

Malaria remains a devastating disease and, with current measures failing to control its transmission, there is a need for novel interventions. A family of proteins that have long been pursued as potential intervention targets are aquaporins, which are channels facilitating the movement of water and other solutes across membranes. We identify an aquaporin in malaria parasites and demonstrate that it is important for completion of *Plasmodium* development in the mosquito vector. Disruption of AQP2 in the human parasite *Plasmodium falciparum* and the rodent parasite *Plasmodium berghei* blocks sporozoite production inside oocysts established on mosquito midguts, greatly limiting parasite infection of salivary glands and transmission to a new host. In vivo epitope tagging of AQP2 in *P. berghei*, combined with immunofluorescence assays, reveals that the protein is localized in vesicle-like organelles found in the cytoplasm of gametocytes, ookinetes, and sporozoites. The number of these organelles varies between individual parasites and lifecycle stages suggesting that they are likely part of a dynamic endomembrane system. Phylogenetic analysis confirms that AQP2 is unique to malaria and closely related parasites and most closely resembles intracellular aquaporins. Structure prediction analyses identify several unusual features, including a large accessory extracellular loop and an arginine-to-phenylalanine substitution in the selectivity filter principally determining pore function, a unique feature among known aquaporins. This in conjunction with the importance of AQP2 for malaria transmission suggests that AQP2 may be a fruitful target of antimalarial interventions.

## Significance

The results reported here uncover a uniquely evolved aquaporin in malaria parasites, which is important for production of sporozoites during the mosquito phase of the parasite lifecycle. Sporozoites are the parasite form responsible for malaria transmission to humans following a mosquito bite. Aquaporins are known transporters of water and other molecules across biological membranes. The aquaporin has unusual structural features and inhabits a vesicle-like organelle inside parasite cells. Since aquaporins can be targeted by drugs, we propose that this aquaporin may be a promising target of future antimalarial interventions.

Malaria, a disease caused by protozoan parasites of the *Plasmodium* genus, remains a significant global health challenge. Progress toward control of malaria has stalled in recent years, primarily due to widespread mosquito resistance to insecticides used in bed nets and indoor residual spraying. Historically, targeting the mosquito vector has proven highly effective in controlling malaria and has even led to disease eradication in some regions. Given the diminishing impact of established interventions, it is of paramount importance to identify new targets for development of novel malaria interventions to control the disease. Here, we describe the identification and characterization of one such putative target: an aquaporin, AQP2, which we show to be important for completion of *Plasmodium* development within the mosquito vector and production of sporozoites, the parasite form responsible for transmission to humans.

Aquaporins are transmembrane proteins that facilitate passive movement of water and other solutes across membranes. They are broadly grouped into exclusive water transporters (orthodox), water channels with solute permeability (e.g., aquaglyceroporins that transport glycerol), and less well-studied intracellular aquaporins (unorthodox or super-aquaporins), although this classification belittles the diversity of aquaporins in terms of both localization and selectivity (1). Aquaporins share a common tertiary structure, with six transmembrane helices arranged in an hourglass pore-forming shape, joined by five extramembrane loops. Two half-helices that dip into either side of the membrane and face each other in the pore center contribute to the pore selectivity and function. Interestingly, aquaporins form tetramers creating a central pore that may additionally transport solutes including ions and small molecules (2–6). While aquaporins on the plasma membrane facilitate exchange between the cell and its environment, intracellular aquaporins facilitate exchange between cytoplasm and organelles. For example, human AQP11 transports hydrogen peroxide into and out of the endoplasmic reticulum (ER) (7), which may explain its essential role in the kidney (8), while AQP1 of the apicomplexan parasite *Toxoplasma gondii* is localized to a plant vacuole-like organelle that serves as a sodium/calcium store and confers stress resistance (9).

*Plasmodium* AQP1 is a well-characterized plasma membrane aquaglyceroporin that conducts water, glycerol, and ammonia at high rates (10–12). It is expressed throughout parasite asexual blood stages (ABS), sporozoites and infected hepatocytes, and provides parasites access to serum glycerol for membrane lipid synthesis (10, 11, 13). While the essentiality of AQP1 to ABS is disputed (11, 14), it is established that the protein is important for progression of hepatic stage infection (13). Interestingly, amino acid residues contributing to AQP1 pore selectivity are identical with those in *Escherichia coli* glycerol facilitator GlpF, which in conjunction with high conservation between the two proteins suggests that AQP1 has been acquired from a prokaryotic ancestor (10, 15).

A second putative aquaporin, AQP2, has been identified in *Plasmodium* genomes and reported to be phylogenetically associated more closely to *T. gondii* AQP1, rather than *Plasmodium* AQP1 (14). We previously performed a reverse genetic screen in the rodent model parasite *Plasmodium berghei* of genes with enriched expression in gametocytes, which identified AQP2 as important for mosquito salivary gland infection (16). Here, we aimed to follow up on these results and characterize AQP2 in both *P. berghei* and the human parasite *Plasmodium falciparum*. Generation and phenotypic characterization of knockout parasites confirmed that AQP2 is important for production of sporozoites in both the rodent and human parasites and for malaria transmission in rodents. Immunofluorescence assays of tagged AQP2 showed that the protein is intracellular, residing in vesicle-like organelles of female gametocytes, ookinetes, and sporozoites. Phylogenetic analysis alongside AlphaFold structure predictions revealed that AQP2 is distinct from AQP1, other aquaglyceroporins and orthodox aquaporins with regard to both its overall architecture and pore selectivity filter. Our results validate AQP2 as a potential transmission blocking target of future antimalarial interventions.

## Results

### AQP2 Is Distinct from AQP1.

AQP2 orthologs are found in all plasmodia with available genome sequences. They encode large aquaporin-like proteins of 603 amino acids in *P. falciparum* (PfAQP2, PF3D7_0810400) and 595 amino acids in *P. berghei* (PbAQP2, PBANKA_1427100). Multiple sequence alignment identified regions of high conservation mostly associated with predicted α-helixes (*SI Appendix*, Fig. S1). One region is substantially divergent between species, both in terms of sequence identity and length: amino acids 110 to 228 in PfAQP2 and 110 to 215 in PbAQP2. Insertions in this region cause AQP2s of human parasites of the subgenus *Plasmodium* (*Plasmodium malariae*, *Plasmodium vivax,* and *Plasmodium knowlesi*) to be considerably longer than other AQP2s. Protein structure predictions made with AlphaFold Monomer v2.0 (17, 18) showed that PfAQP2 and PbAQP2, like all aquaporins, have six full transmembrane helices and two half helices, connected by five loops (*SI Appendix*, Fig. S2). The variable region corresponds to extracellular loop A that lies between helixes 1 and 2. Loop A is highly charged, with roughly 40% of amino acids carrying either a positive or negative charge. Intracellular loop D is longer than extracellular loop C, the inverse of loop lengths of *Plasmodium* AQP1. The characteristic aquaporin pore forming Asparagine-Proline-Alanine (NPA) motifs are present in all AQP2s, in half-helices 1 and 2 that are part of loops B and E, respectively. While the first NPA motif is present as a canonical NPA, the second motif is found as Asparagine-Proline-Methionine (NPM) in all species except *Plasmodium gallinaceum* which harbors an A/M>L substitution. Finally, in all AQP2s, the canonical arginine residue that determines the selectivity filter, invariably found directly adjacent to the second NPA motif, is replaced with phenylalanine (R > F).

To understand the evolutionary history of *Plasmodium* AQP2s, we performed a phylogenetic analysis of the conserved pore-forming regions of AQP2s and 66 other aquaporins from 21 other species across all domains of life, including *Homo sapiens* and *Anopheles gambiae*—the vertebrate and mosquito hosts of *P. falciparum*, respectively (*SI Appendix*, Fig. S3). A clear clustering of aquaporins was observed principally by the type of pore formed, whether orthodox, solute transporters including aquaglyceroporins, or unorthodox. *Plasmodium* AQP2s form a well-defined clade together with an aquaporin of the closely related parasite *Hepatocystis*, which appears to be related to intracellular *T. gondii* AQP1 (TgAQP1). A clade involving *A. gambiae* AQP7 and the unorthodox human aquaporins AQP11 and AQP12 are the closest to *Plasmodium* AQP2s and TgAQP1, although this relation lies deep in the root of the tree and is not supported by bootstrapping. *Plasmodium* AQP1s form a separate clade together with human aquaglyceroporins and *E. coli* glycerol facilitator GlpF, as previously described (10).

From these data, we concluded that AQP2 most closely resembles intracellular, unorthodox aquaporins, with their pore-forming sequences closely related to *Hepatocystis* aquaporin and, albeit less so, to the intracellular, vacuole-localized TgAQP1. The R > F substitution in the AQP2 selectivity filter is unique to *Plasmodium*, however.

### AlphaFold Structure Predictions Reveal Unusual AQP2 Pore and Selectivity Filter Features.

We used AlphaFold Monomer v2.0 (17, 18) to study the putative three-dimensional structure of PfAQP2 and PbAQP2 (UniProt C0H4T7 and A0A509AUY5, respectively). The results showed that the highly conserved pore-forming helices have the highest confidence structure predictions (*SI Appendix*, Fig. S4). However, helix 6 alongside loop E of PbAQP2 has low confidence prediction and does not correctly fold into the core as expected. Therefore, we proceeded with analysis of the PfAQP2 structure as the absence of helix 6 from the PbAQP2 structure may result in unintended changes to the pore. Nevertheless, all the observations below match those also made for PbAQP2 (*SI Appendix*, Fig. S5).

**Fig. 1.**
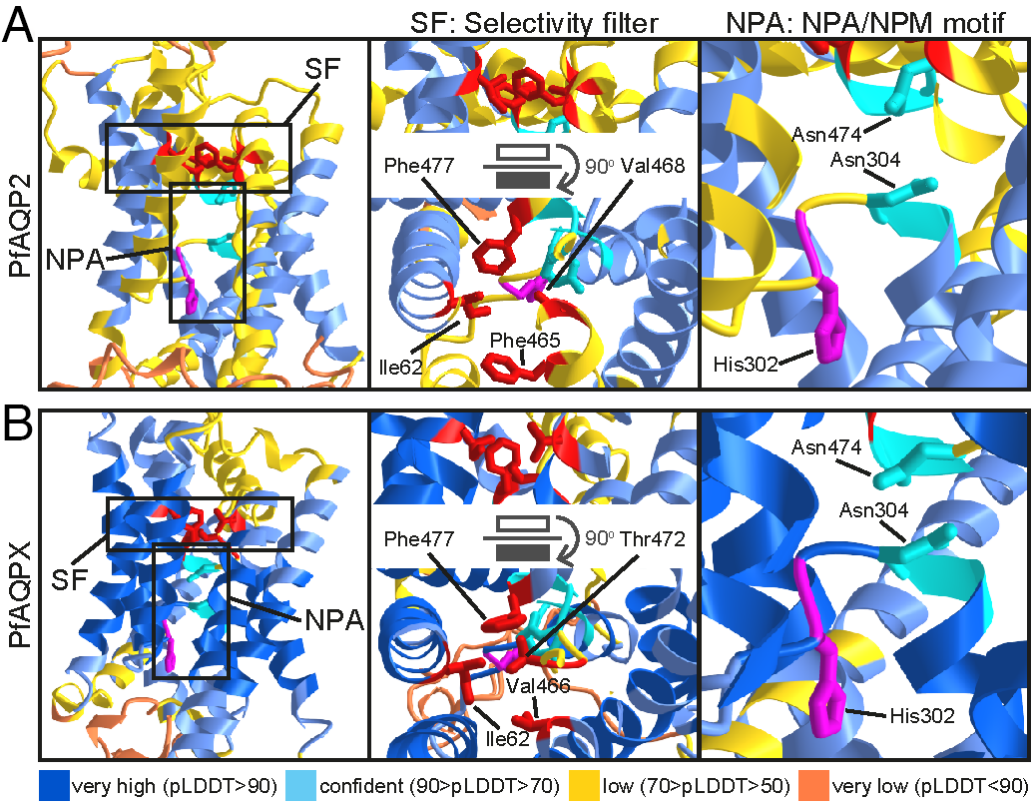
Predicted structure of PfAQP2 pore and selectivity filter. (*A*) PfAQP2. (*B*) Chimeric PfAQPX in which PfAQP2 loops A-E are replaced with corresponding loops of previously resolved PfAQP1 structure. Structures are predicted with AlphaFold Monomer v2.0 on the CoLab server. Top ranking models are shown. Structures are colored by confidence level as shown in the key below the figure. Residues contributing to the selectivity filter are shown in red and those contributing to the NPA/NPM motif are shown in cyan, while the histidine residue participating in the pore is shown in magenta. SF is the selectivity filter, NPA is the Asparagine–Proline–Alanine motif, and NPM is Asparagine–Proline–Methionine. White and black rectangles indicate the orientation of the top relative to the bottom frame by a 90-degree rotation.

Overall, the AlphaFold PfAQP2 structure provides high-confidence predictions of the pore-forming regions including the selectivity filter and downstream NPA/NPM motifs (Fig. 1*A*). The four residues constituting the constriction at the selectivity filter are Ile62 (helix 1), Phe465 (helix 5), Val468 (helix 5) and Phe477 (half helix 2). While Phe477 on the site canonically occupied by an arginine residue does not appear to affect the formation of the pore, the presence of two aromatic phenylalanine residues opposite each other is a major difference from canonical aquaporins and may pose a restriction to the size of the pore. The isoleucine residue is contributed to the pore by helix 1 as opposed to helix 2 as in most other aquaporins, causing a substantial tilt of AQP2 in the membrane axis, but this does not change the predicted structure of the pore. The NPA and NPM motifs contributed by half-helices 1 and 2, respectively, meet one another in the pore. Interestingly, the central pore is mostly nonpolar; however, a positively charged histidine residue (His302) found near the interior side of the NPA/NPM motif places a positively charged residue close to the pore center.

To generate more high-confidence pore predictions, we generated a chimeric sequence with extramembrane PfAQP2 loops A-E replaced with the shorter loops of PfAQP1 that has a resolved tertiary structure (15). The chimeric protein is referred to as PfAQPX. Folding predictions using AlphaFold on the CoLab server indeed generated much higher confidence predictions of PfAQPX pore structure (Fig. 1*B*). While most PfAQP2 pore features and structure were preserved in PfAQPX, Phe465 was rotated away to no longer participate in the filter and instead replaced by Thr472 from half helix 2 that is a more typical selectivity filter residue contribution. All the rest of the amino acid residues remained the same as in the PfAQP2 structure, including Phe477.

### PfAQP2 and PbAQP2 Are Important for Sporozoite Production in the Oocyst.

**Fig. 2.**
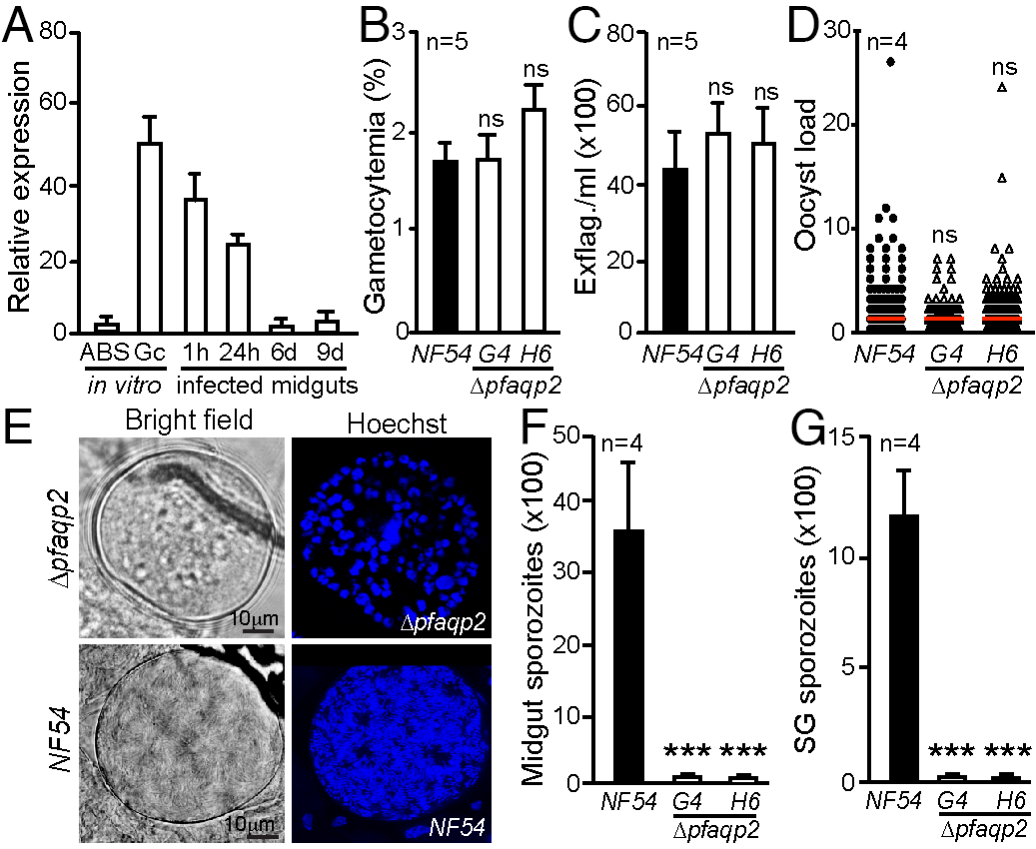
Phenotypic characterization of *P. falciparum* AQP2. (*A*) QRT-PCR of AQP2 transcripts in in vitro cultured ABS and mature gametocytes (Gc) and in in vivo infections of (*A). coluzzii* midguts at 1 h, 24 h, 6 d, and 9 d pbf. Relative expression was calculated for each sample relative to in vitro and in vivo internal calibrator reference values using EF1α as a housekeeping gene. Mean and SEM values from three independent replicates are presented. (*B*) Percentage gametocytemia in total parasite numbers in in vitro gametocyte culture. Mean and SEM values from five independent replicates are shown. (*C*) Number of exflagellation centers per 1 mL of in vitro gametocyte culture. Mean and SEM are shown. (*D*) Oocyst load in mosquito midguts 9 d pbf. Medians from pools of four replicates are presented (red lines). (*E*) Brightfield and Hoechst imaging of oocysts 11 d pbf. (The scale bar is 10 μm.) (*F*) Midgut and (*G*) salivary gland sporozoites enumerated using a hemocytometer with mean and SEM presented. In all panels, results from *NF54* control and two independent *Δpfaqp2* parasite lines (G4 and H6) are presented. Statistical analysis is done with ANOVA for data in panels (*B* and *C*) and (*F* and *G*) and the Kruskal–Wallis test for data in panel (*D*). ns means not significant and *** shows *P* < 0.001.

Our earlier work has shown that PbAQP2 expression is higher in gametocytes than ABS (16), and this is confirmed by a separate single-cell transcriptomic study (19). We sought to study the expression of AQP2 in *P. falciparum* by quantitative real-time RT-PCR (qRT-PCR). In ABS, there is very low expression of AQP2, in contrast to in vitro cultured gametocytes that express the highest amount of AQP2 in all stages assayed (Fig. 2*A*). In *Anopheles coluzzii* infections, highest AQP2 expression was detected in the blood bolus 1 h pbf (post blood feeding), a signal that most likely derives from gametocytes, which remains high 24 h pbf, a timepoint that also encompasses mature ookinetes. Following establishment of oocysts, AQP2 transcription appeared to be minimal 6 d pbf, but a slight increase was observed 9 d pbf, suggesting possible re-expression in maturing sporozoites.

We generated a PfAQP2 deletion mutant via CRISPR/Cas9 and single homologous recombination (*SI Appendix*, Fig. S6*A*). Successful integration of a selectable marker in *P. falciparum* NF54 and limiting dilution cloning, confirmed by diagnostic PCR, led to the generation of two successful *Δpfaqp2* clones (G4 and H6; *SI Appendix*, Fig. S6*B*). The capacity of *Δpfaqp2* to form gametocytes was assessed 14 d after induction of in vitro cultured ABS. No significant difference was observed between NF54 and the *Δpfaqp2* lines in the establishment of mature stage V gametocytes (Fig. 2*B*). Next, we performed in vitro exflagellation assays to assess the capacity of *Δpfaqp2* to produce male gametes by counting exflagellation centers. Again, no difference was observed between the *NF54* control and the two *Δpfaqp2* lines (Fig. 2*C*).

Oocyst presence on the midguts of *A. coluzzii* 9 d pbf on in vitro gametocyte cultures was comparable between *NF54* control and *Δpfaqp2* lines, both in terms of load, i.e., number of oocysts per midgut, and prevalence, i.e., proportion of mosquitoes harboring oocysts (Fig. 2*D* and *SI Appendix*, Table S1). However, imaging oocysts 11 d pbf indicated that nuclear division into daughter nuclei for budding sporozoites was less advanced in *Δpfaqp2* compared to *NF54* controls (Fig. 2*E*). Most, albeit not all, *Δpfaqp2* oocysts exhibited larger nuclei and smoother appearance compared to *NF54* oocysts, lacking the characteristic convolutions associated with budding sporozoites. Indeed, mechanical disruption of oocysts followed by counting of sporozoite numbers using hemocytometer confirmed almost total absence of mature sporozoites from *Δpfaqp2* oocysts 11 d pbf compared to thousands of sporozoites per mosquito harboring *NF54* oocysts (Fig. 2*F* and *SI Appendix*, Table S2). Next, we enumerated sporozoites in mosquito salivary glands 18 d pbf. The results corroborated the oocyst findings, as only very few *Δpfaqp2* compared to thousands of *NF54* sporozoites were detected per mosquito (Fig. 2*G* and *SI Appendix*, Table S2). These data led us to conclude that loss of *P. falciparum* AQP2 results in a dramatic reduction in development of mature sporozoites but has no impact on any of the preceding stages.

We extended our investigation to AQP2 of *P. berghei* as this parasite allows more detailed analysis and mosquito-to-mouse transmission experiments. *P. berghei* mutant lines carrying a disrupted version of AQP2 were generated using a *Plasmo*GEM vector that replaced 70% of the AQP2 coding sequence with the *human DHFR* (*hDHFR)/yFCU* pyrimethamine resistance cassette in the *c507* GFP-expressing wild-type line (20). Integration of the disruption cassette and gene deletion in clonal *Δpbaqp2* was confirmed by PCR (*SI Appendix*, Fig. S7*A*).

**Fig. 3.**
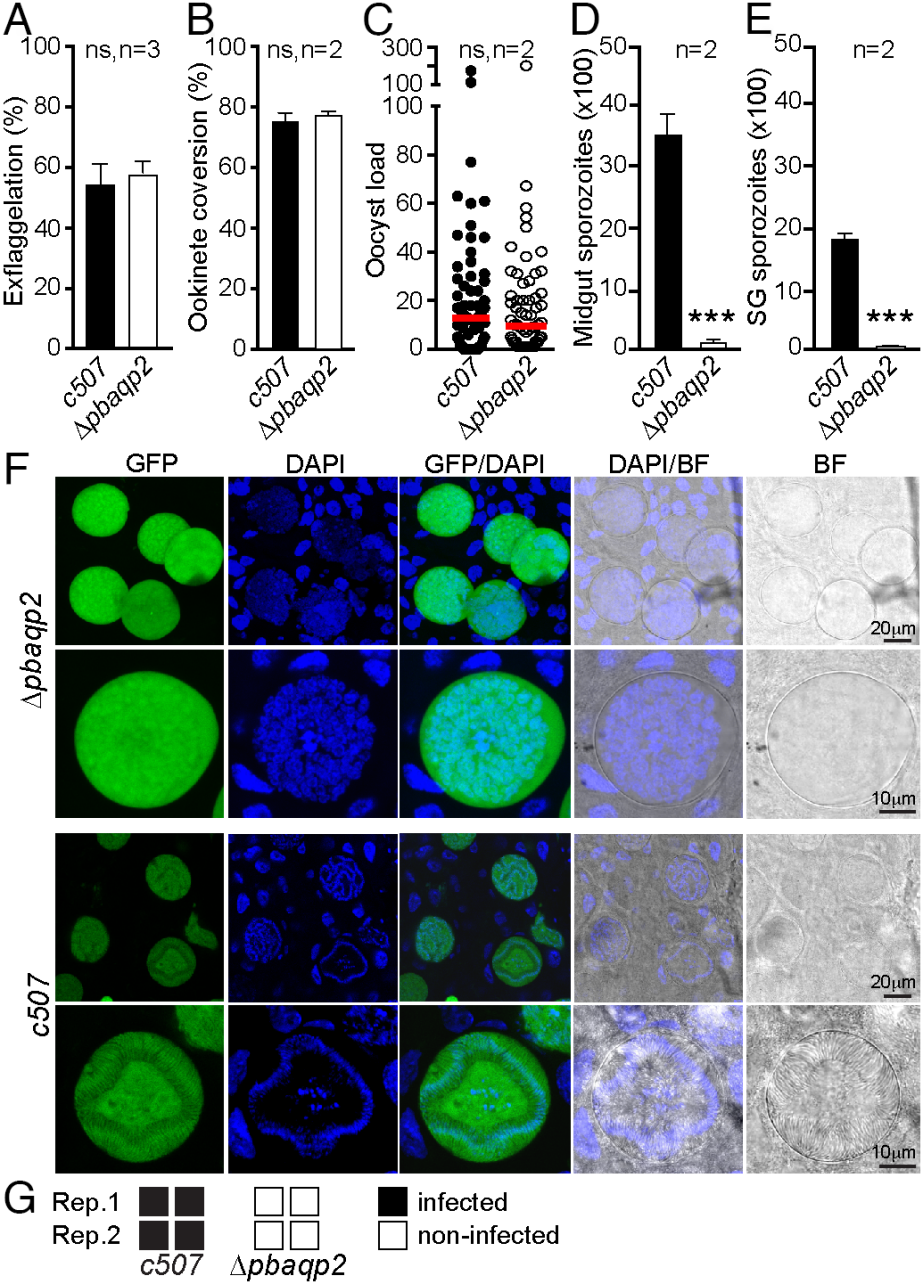
Phenotypic characterization of *P. berghei* AQP2. (*A*) Percentage in vitro exflagellation of male gametocytes. (*B*) Female gamete to ookinete conversion. (*C*) Oocyst load in the midguts of *A. coluzzii* mosquitoes enumerated 8 d pbf. (*D*) Number of sporozoites in mechanically ruptured oocysts 15 d pbf. (*E*) Number of sporozoites in mosquito salivary glands 21 d pbf. SG is the salivary gland. Graphs *A*, *B*, *D*, and *E* depict mean and SEM with statistical analysis done with Student’s *t* test. Graph *C* shows median (red lines) and distribution with statistical analysis done with the Mann–Whitney *U* test. n is the number of independent biological replicates; ns is not significant; *** is *P* < 0.001. (*F*) Fluorescence microscopy images of GFP-expressing *Δpbaqp2* and *c507* control oocysts at day 15 pbf in *A. coluzzii* midguts. Brightfield (BF) and DAPI staining are shown. (*G*) Bite-back assays where mosquitoes were allowed to feed on C57/BL6 mice 21 d pbf. Each mouse is represented by a square. Rep: replicate infection.

Male gametogenesis, determined as the number of exflagellation events per male gametocytes, was comparable between *Δpbaqp2* and the parental *c507* control line (Fig. 3*A*), as was the capacity of female *Δpbaqp2* gametocytes to form ookinetes (Fig. 3*B*). Next, we assessed the ability of *Δpbaqp2* to form oocysts following feeding of *A. coluzzii* mosquitoes on infected mice. Oocyst enumeration in mosquito midguts 8 d pbf showed no difference in oocyst load and prevalence between *Δpbaqp2* and *c507* control parasites (Fig. 3*C* and *SI Appendix*, Table S3).

We investigated the ability of *Δpbaqp2* oocysts to produce sporozoites by quantifying sporozoites in mechanically ruptured oocysts 15 d pbf and in the mosquito salivary glands 21 d pbf. The results were consistent with those obtained for *Δpfaqp2* parasites: both *Δpbaqp2* oocyst and salivary gland sporozoite numbers were severely reduced compared to the *c507* controls (Fig. 3 *D* and *E* and *SI Appendix*, Table S4). DAPI staining of *Δpbaqp2* oocysts at day 15 pbf revealed large and disorganized nuclei, in contrast to the highly organized nuclei of *c507* oocysts, which is characteristic of sporozoites that have budded off from the central sporoblastoid body (Fig. 3*F*).

To determine whether this phenotype indicates a complete inability of *Δpbaqp2* oocysts to undergo sporulation or a delay in sporozoite production, we repeated *A. coluzzii* infections using both c507 controls and *Δpbaqp2* parasites and quantified midgut sporozoite numbers at days 15, 16, 18, and 20 pbf. The sporozoite counts in *Δpbaqp2* oocysts not only failed to recover compared to the controls but also completely diminished by day 18 pbf (*SI Appendix*, Fig. S8). This suggests that the small number of sporozoites produced at earlier time points may have been released into the hemocoel.

Last, we conducted bite-back assays on highly susceptible C57/BL6 mice to determine whether the small number of *Δpbaqp2* sporozoites in the mosquito salivary glands could still be transmitted between vertebrate hosts through mosquito bites. The infected mosquitoes used for these assays were derived from the same two independent replicate infections presented in *SI Appendix*, Table S4. For each replicate, two groups of 25 to 30 *c507* control and two groups of 25 to 30 *Δpbaqp* infected mosquitoes were allowed to feed on a single mouse 21 d pbf. The mice were monitored daily for 14 d after the mosquito bites. None of the mice bitten by mosquitoes infected with the *Δpbaqp2* parasites developed parasitemia, indicating that transmission was terminated (Fig. 3*G*).

### AQP2 Resides in Vesicle-Like Organelles of Ookinetes and Midgut Sporozoites.

**Fig. 4.**
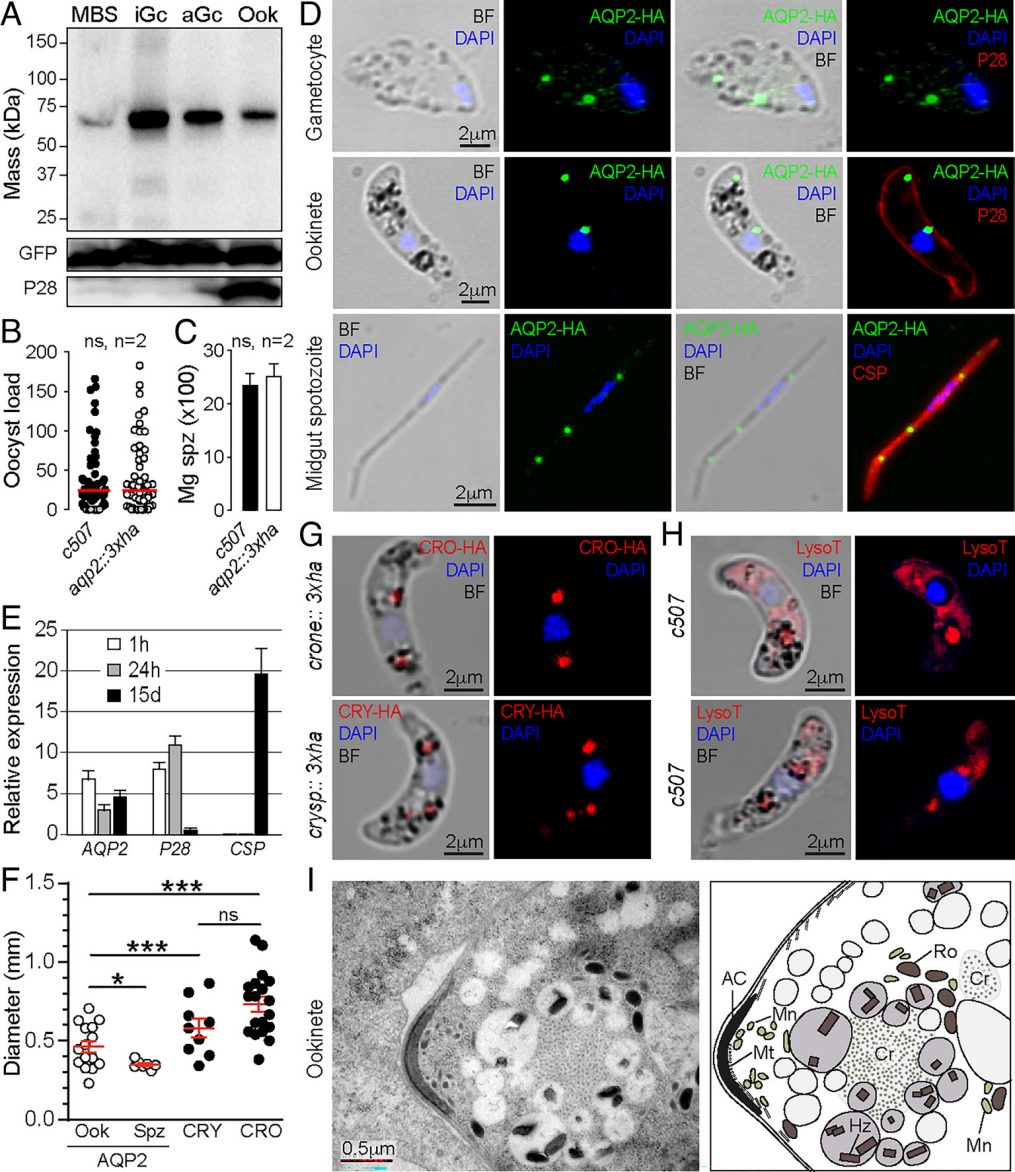
PbAQP2 expression and localization. (*A*) Western blot analysis using α-HA antibody on cell lysates of *aqp2::3xha* parasites under reducing conditions. GFP and P28 are used as loading and stage-specific controls, respectively. MBS; mixed blood stages. iGc: inactivated gametocytes, aGc: activated gametocytes, Ook: ookinetes. (*B*) Distribution and median of oocyst load in (*A). coluzzii* midguts mosquitoes infected with the *aqp2::3xha* line, 9 d pbf. Statistical analysis is conducted using the Mann–Whitney *U* test. n: number of replicates, ns: not significant. (*C*) Number of *aqp2::3xha* sporozoites in oocysts 15 d pbf. Whiskers represent SEM. Statistical analysis is conducted using Student’s *t* test. (*D*) IFAs of *aqp2::3xha* purified gametocytes, in vitro cultured ookinetes, and sporozoites from infected *A. coluzzii* midguts. AQP2::3xHA (green) and P28 (red, ookinetes) or CSP (red, sporozoites) are detected using antibodies. DNA stained with DAPI (blue). BF; brightfield. (*E*) QRT-PCR of *PbAQP2* transcripts in infected *A. coluzzii* at different time points pbf. Relative expression calculated relative to EF1α housekeeping control. P28 and CSP are used as controls. Mean and SEM values from three replicates are presented. (*F*) Diameter comparison of AQP2::3xHA-stained vesicles in ookinetes (Ook) and sporozoites (Spz) with crystalloids determined via CRYSP::3xHA (CRY) and CRONE::3HA (CRO) proteins. (*G*) IFAs of *crone::3xha* and *crysp::3xha* in vitro cultured ookinetes using anti-HA antibody (red). DNA stained with DAPI (blue). BF; brightfield. (*H*) Live staining of in vitro produced ookinetes with LysoTracker. DNA stained with DAPI (blue). BF: brightfield. (*I*) Electron micrograph of the ookinete in the *A. coluzzii* midgut (*Left*) and a schematic showing the various observations (*Right*). AC: apical complex, Cr: crystalloid, Hz: hemozoin, Mn: micronemes, Mt: microtubules, Ro: rhoptries.

We tagged PbAQP2 at its C terminus with a 3x human influenza hemagglutinin (3xHA) epitope via double cross-over homologous recombination in the *c507* line to study protein expression. The transgenic parasite line was named *aqp2::3xha* (*SI Appendix*, Fig. S7*B*). Western blot analysis of *aqp2::3xha* parasite protein extracts using an antibody against the HA epitope confirmed that recombinant AQP2::3xHA is produced at the expected ca. 73 kDa size predominantly in gametocytes—already prior to gametogenesis activation—and, at a lower abundance, in mature ookinetes (Fig. 4*A*). Protein traces observed in mixed blood stages are thought to derive from gametocytes. Infections of *A. coluzzii* mosquitoes revealed that the *aqp2::3xha* line produces comparable infection load and prevalence (82.1% vs. 81.1%) to the parental c507 parasite line 9 d pbf (Fig. 4*B*). Furthermore, similar numbers of midgut sporozoites were observed 15 d pbf between *aqp2::3xha* and c507 controls (Fig. 4*C*), and bite-back assays conducted 21 d pbf confirmed successful transmission of *aqp2::3xha* between mice through mosquito bites (2 of 2 mice). Taken together, these findings demonstrate that the addition of the HA tag does not compromise the functionality of AQP2.

Indirect immunofluorescence assays of *aqp2::3xha* parasites revealed that AQP2::3xHA specifically localized to vesicle-like structures in the cytoplasm of gametocytes, ookinetes, and midgut sporozoites (Fig. 4*D*). The number and appearance of these structures varied, with gametocytes typically having 1 to 2 large spots and several smaller ones, ookinetes displaying 2 to 3 spots (occasionally 1 or 4), and sporozoites exhibiting 2 to 3 spots (Fig. 4*D* and *SI Appendix*, Fig. S9). The pronounced signal of AQP2::3xHA in midgut sporozoites prompted us to investigate the expression of *aqp2* in this lifecycle stage. Indeed, qRT-PCR confirmed strong expression of *aqp2* in infected *A. coluzzii* midguts 15 d pbf (Fig. 4*E*). Interestingly, salivary gland AQP2::3xHA sporozoites did not exhibit these spots (*SI Appendix*, Fig. S9).

In ookinetes, the localization pattern of AQP2::3xHA resembled that of the crystalloid, an elusive organelle exclusive to ookinetes and young oocysts. Although these spots were often seen adjacent to the hemozoin crystals (the waste product of hemoglobin metabolism and a hallmark of the ABS food vacuole and the crystalloid), they did not colocalize with the hemozoin or locate among the hemozoin crystals where the crystalloid is typically found. Notably, the diameter of these structures in ookinetes (0.2 to 0.7 μm) was smaller than that of crystalloids (0.3 to 1.15 μm), as determined by IFAs of the recently identified crystalloid proteins CRYSP and CRONE (16) (Fig. 4 *F* and *G*). In sporozoites, where hemozoin is not typically observed, the diameter of these structures was more uniform (0.25 to 0.4 μm).

We hypothesized that the AQP2::3xHA spots corresponded to vesicles within the parasite endomembrane system. We examined the nature of these vesicles by staining of ookinetes with LysoTracker, a fluorescent dye that selectively labels acidic cellular compartments. As this staining was conducted on live parasites, we were unable to simultaneously track the localization of AQP2::3xHA. The results revealed intense LysoTracker staining of crystalloids and other cellular compartments, typically adjacent to the crystalloids, likely including vesicles containing AQP2::3xHA (Fig. 4*H*). Vesicles housing hemozoin did not exhibit any detectable LysoTracker staining.

To gain a clearer understanding of the ookinete cellular compartments associated with the crystalloid, we conducted electron microscopy analysis on infected *A. coluzzii* midguts 24 h pbf. Consistently, our observations revealed that crystalloids are enveloped by multiple vesicles of varying sizes (Fig. 4*I*). While many of these vesicles contain hemozoin, indicating their involvement in hemoglobin metabolism and waste management, there are also several vesicles that lack hemozoin, likely including those housing AQP2. These findings suggest that the crystalloid may serve as a nucleating compartment for vesicles within the parasite endomembrane system, which have diverse functions likely associated with the crystalloid.

## Discussion

Our study reveals that *Plasmodium* AQP2 is an intracellular aquaporin localized within vesicle-like structures found in gametocytes, ookinetes, and sporozoites. Already in 1962, Garnham and coworkers described ookinete cellular bodies with a more regular shape and uniform structure than crystalloids, which resembled lysosomes (21). While the crystalloid is now known to store proteins essential for oocyst development (22), these other cellular bodies have remained unstudied. Based on the appearance, size, and varying number of the AQP2-inhabiting structures in individual parasites and lifecycle stages, we propose that they are part of a dynamic parasite endomembrane system and may correspond to these lysosome-like bodies.

Notably, one of the closest phylogenetic relatives of AQP2, the intracellular *T. gondii* AQP1 resides in a multivesicular organelle that exhibits similarities to the plant-like vacuole (PLV) (9). Plants have two types of vacuoles: the lytic vacuole, which contains hydrolytic enzymes and functions as a digestive organelle similar to lysosomes, and the protein-storage vacuole (23). Intriguingly, plant vacuoles contain Tonoplast Intrinsic Proteins (TIP) aquaporins (24), which are related to *T. gondii* AQP1 (25). Therefore, we speculate that while the crystalloid is equivalent to the plant protein-storage vacuole, the AQP2-containing bodies are part of a vesicular endomembrane system equivalent to the lytic vacuole. Within these organelles, AQP2 could play a role in water or energy storage or trafficking of essential solutes, similar to the functions observed in *T. gondii* AQP1 and human AQP11 (7–9, 25), thereby serving an essential role in homeostasis by regulating the pH, osmolarity or redox status. These organelles are hypothesized to be functionally related to the crystalloid as they are typically found next to each other, with the crystalloid appearing to serve a nucleating role for both AQP2 and hemozoin housing vesicles. It is worth noting that although both the crystalloid and the AQP2-containing vesicles are prominent in the ookinete, the essential functions of their resident proteins are detected days later in the maturing oocyst.

The detection of AQP2-harboring vesicles in the sporozoite presents an additional challenge, as there is no reference of lysosomes in the sporozoite. A number of prominent vesicles in the region between the nucleus and apex, named micropores, have been reported in early studies (26–29). Their increase in numbers late in sporozoite development made authors suggest an important function in parasite maturation. Micropore vesicles are up to 0.25 μm in diameter, which matches the size of AQP2-harboring vesicles (fluorescence measurements may inflate vesicle size), filled with granular material similar to those present in the oocyst matrix; therefore, they have been interpreted as endocytic vesicles. Interestingly, endocytic vesicles with similar contents have been described in *T. gondii* (30). Although these may be related to the PLV, no such connection has been made in the literature. Indeed, a later study has concluded that it is impossible to determine whether these vesicular structures are being trafficked in or out of the parasite (31).

Like *T. gondii* AQP1, *Plasmodium* AQP2 exhibits an amino acid substitution at the critical aromatic/arginine (ar/R) region of the pore. In TgAQP1, arginine is replaced by a valine (25), a substitution characteristic of plant TIP-like aquaporins, and in AQP2 arginine is replaced by phenylalanine. While both residues are hydrophobic, phenylalanine is larger and aromatic compared to valine. Phenylalanine is commonly found in orthodox aquaporin and aquaglyceroporin filters but not in the place of the canonical arginine, making it a unique feature of AQP2. Furthermore, the presence of two phenylalanine residues in the selectivity filter would make the AQP2 pore highly hydrophobic. However, it is important to note that this predicted structure derives from unconfident AlphaFold predictions, highlighting the need for future structural studies on purified AQP2. In fact, when all the loops of AQP2 are replaced with those of AQP1, resulting in a more confident AlphaFold prediction of the central pore region, one phenylalanine residue in the selectivity filter is replaced by threonine, a smaller and polar residue, leaving only the unusual ar/R-substituting phenylalanine in the pore. Notably, while the replaced phenylalanine appears not to protrude directly into the pore but is rotated away from its central axis, threonine extends further toward the pore center, resulting in a tighter constriction. The presence of a phenylalanine or threonine in the AQP2 pore would likely alter solute selectivity modifying both the polarity and diameter of the pore. Nevertheless, both possible filters are mostly nonpolar and lack charged residues. Interestingly, all predictions indicate that isoleucine is contributed to the selectivity filter by helix 1 instead of helix 2 as in other aquaporins with resolved structures. This structural arrangement gives AQP2 a substantial tilt in the membrane axis. Overall, *Plasmodium* AQP2 is highly likely not an orthodox aquaporin.

In addition to its divergent pore, *Plasmodium* AQP2 possesses several intriguing features in its extramembrane loop regions, particularly in extracellular loop A, which is considerably larger in size than other aquaporins, although its length varies among *Plasmodium* orthologs. Loop A is typically short in known aquaporins, such as the 6-amino acids loop A in AQP1 (10, 15). An extended loop A is found in plant PIPs, where it appears to be involved in disulfide bonding of heterotetramers and can form a cap gating the central pore (32). In PIP2;1, the negative charges present in loop A are suggested to play a role in ion conduction through the pore. Although PfAQP2 or PbAQP2 loops do not contain cysteines, they do have a high number of charged residues. For example, PfAQP2 loop A has 39 positively charged and 44 negatively charged of a total of 211 residues. Therefore, the net-negative charge of loop A may regulate cation conductance through a putative AQP2 tetramer, like PIP2;1, be involved in gating of either the monomer or central pore, or facilitate interaction with other regulatory proteins, akin to *Saccharomyces cerevisiae* aquaglyceroporin FPS1. In FPS1, unusually large N- and C-terminal extensions are used to regulate the channel permeability in response to stress by recruiting regulatory proteins such as kinase Hog1 and regulators of glycerol channel Rgc1 and Rgc2 (33, 34).

Regardless of its exact function, AQP2 plays an important role in sporozoite production within the oocyst, and its loss of function leads to a limited number of sporozoites in the salivary glands. In the rodent model, we show that AQP2 is required for disease transmission. A recent study in another rodent parasite, *Plasmodium yoelii*, has demonstrated a nonlinear relationship between salivary gland sporozoite load and mosquito infectiousness probability, with mosquitoes harboring over 10,000 sporozoites in their salivary glands being significantly more likely to initiate an infection (35). This leads us to propose that AQP2 could be a promising target for transmission-blocking drugs, given its presence in blood-stage gametocytes, or other antimalarial interventions in the vector. Aquaporin channels have garnered interest as potential targets for various diseases, such as cancer, metabolic disorders, and edema, and the widespread occurrence, significance, and divergence of aquaporins in protozoan parasites make them potential targets for controlling infectious diseases (36). Notably, a recent study has reported the inhibition of *P. falciparum* growth by blocking AQP1 with the sweetener erythritol (37). The unique evolutionary history of *Plasmodium* AQP2 and the divergence of its selectivity filter from known aquaporins present an attractive opportunity for developing small molecules that specifically target AQP2 with minimal cross-reactivity to aquaporins in host and vector species.

## Materials and Methods

### Multiple Sequence Alignment.

AQP2 orthologs from 8 *Plasmodium* species were identified and amino acid sequences retrieved using PlasmoDB: PF3D7_0810400, PPRFG01_0811800, PmUG01_14045900, PVP01_1429900, PKNH_1430300, PBANKA_1427100, PY17X_1429200, and PGAL8A_00525700. Multiple sequence alignments were performed with T-Coffee v11.00 (38), and the resulting alignments were presented and annotated using ESPript3.0 (39).

### Phylogenetic Analysis.

Pore-forming motifs were identified, and the 60 amino acids surrounding each motif were extracted for multiple sequence alignment performed in R using the msa package (v1.26). Distance matrices were calculated by the JC69 method using ape (v5.6.2). Phylogenetic trees were estimated using the Neighbor-Joining method (40) and generated using the ggtree package (v3.2.1), adapted from code produced previously (41). Bootstrap values were derived from 1,000 iterations using ape (v5.6.2).

### Prediction of Tertiary Structures Using AlphaFold.

AlphaFold Monomer v2.0 (17, 18) was used to predict protein tertiary structures. For folding of PfAPQX, sequences were first adapted manually, and tertiary structures were predicted using the ColabFold server, which uses sequence alignments generated by MMseqs2 and HHsearch followed by AlphaFold Monomer v2.0 to fold custom sequences (42). 3D structures were visualized and colored in iCn3D v3.18.1 (43).

### Prediction of Membrane Topology.

Membrane-spanning helices presented in tertiary structures produced by AlphaFold and visualized in iCn3D were cross-referenced with predicted membrane-spanning sequences predicted by PPM2.0 (44). Predicted membrane topology was visualized, annotated, and presented using Protter (45).

### Mosquito Maintenance.

*A. coluzzii* mosquitoes of Ngousso strain were maintained in an insectary at 26 °C and 70% relative humidity, with a 12-h day/night cycle with 30-min dawn/dusk transitions, respectively.

### *P. falciparum* Culturing.

NF54 *P. falciparum* was maintained in 10 mL human red blood cell (A+) cultures at 5% hematocrit (HTC; Cambridge Biosciences) in complete medium (CM: RPMI-1640-R5886, hypoxanthine (0.05 g/L), L-glutamine (0.3 mg/L), and 10% sterile human A+ serum (Interstate Blood Banc Inc.). Human serum was batch tested for compatibility with gametocyte production. Asexual parasite cultures were maintained by dilution once weekly to limit parasitemia to <10%, and media were refreshed daily. Cultures were fumigated with malaria gas mixture (5% O_2_, 5% CO_2_, and 90% N_2_) and maintained in static incubators at 37 °C.

### *P. falciparum* Mosquito Infections.

*A. coluzzii* Ngousso mosquitoes separated into pots the day prior to feeding for acclimatization were infected with *P. falciparum* by standard membrane feeding as previously described (46). Briefly, gametocytes were induced from asexual *P. falciparum* cultures by establishment of 1% parasitemia cultures in reduced conditioned media, resulting in a 5% HTC culture in 8 mL volume. Cultures were maintained in reduced volume at 37 °C and in malaria gas atmosphere for 14 d until mosquito feed. Parasite cultures were pelleted and resuspended with fresh RBCs (200 µ per culture flask) and equal volume of human A+ serum (Interstate Blood Banc Inc.). Infected blood was loaded into membrane feeders thinly covered by Parafilm connected to a water bath at 37 °C, and feeders were introduced to the mosquito pots. Feeding was enabled for 10 min before mosquitoes were removed and stored in secondary containment in an incubator at 27 °C and 70% relative humidity to establish infection. Blood fed mosquitoes were kept in the dark for 48 h postfeed with no sugar feed provided to ensure survival of only fed females. After this period, mosquitoes were provided with 10% sucrose until dissection.

### Transcriptional Profiling Using qRT-PCR.

Total RNA was isolated using the Trizol® reagent (ThermoFisher). Quantitative RT-PCR reactions were performed on cDNA samples using SYBR-Green premix (Applied Biosystems) in an Applied Biosystems Real-Time PCR System and gene-specific primers (*SI Appendix*, Table S5). PfAQP2 transcript levels were normalized against EIF1α transcripts. PbAQP2 transcript levels were normalized against transgenic GFP transcripts.

### Generation of AQP2-Dis-pDC2 Plasmid for Disruption of PfAQP2.

The pDC2 plasmid kindly provided by Marcus Lee (Sanger Institute) was digested and linearized with BbsI (New England Biosciences) to create TATT/AAAC overhangs downstream of the U6 promoter for insertion of the gRNA sequence. The gRNA-encoding DNA oligomers (410 to 432 bp of PfAQP2 coding sequence) with appended TATT/AAAC (*SI Appendix*, Table S5) were annealed and phosphorylated with T4 polynucleotide kinase enzyme prior to ligation into the linearized plasmid. Following gRNA insertion, the plasmid was prepared for insertion of donor DNA sequences by Gibson Assembly. All PCR reactions described were performed with CloneAmp HiFi polymerase (Takara) and the specified primers in an Applied Biosystems Thermocycler according to the manufacturer’s instructions. First, the hDHFR resistance cassette was cloned from pDC2 using the P5/P6 primer pair (*SI Appendix*, Table S5). Homology arms of 863 bp upstream and 593 bp downstream of the gRNA target site were cloned from *P. falciparum* genomic DNA (gDNA) using primer pairs P1/P2 and P3/P4, respectively, incorporating overlap sequences for Gibson Assembly into the PCR product. The pDC2 backbone was linearized by restriction enzyme digest with ApaI/AatII (New England Biosciences). DNA fragments were assembled using the NEBuilder kit (New England Biosciences) according to the manufacturer’s instructions, reconstituting the pDC2 plasmid with AQP2 5′ and 3′ homology regions flanking hDHFR.

### Transfection of *P. falciparum* by Electroporation.

Plasmids were amplified in *E. coli* by transformation into competent cells followed by liquid culture of clonal colonies; plasmids were then isolated using an endotoxin-free QIAGEN maxiprep kit. Fifty µg of each plasmid per transfection was ethanol precipitated overnight [2.5 volumes 100% ethanol and 1/10 volumes sodium acetate (3M, pH5.2), −20 °C]. Pellets were washed in 70% ethanol and resuspended in sterile TE buffer (QIAGEN). Fifty µg of fully dissolved plasmid was taken into a final volume of 50 µL sterile TE and added to 350 µL sterile incomplete cytomix (120 mM KCl, 0.15 mM CaCl_2_, 2 mM EGTA, 5 mM MgCl_2_, 10 mM K_2_HPO_4_, 10 mM KH_2_PO_4_, and 25 mM HEPES pH7.6). Five mL *P. falciparum* cultures at 5% hematocrit were synchronized with 5% sorbitol in advance of transfection; cultures were established to achieve 5 to 6% early ring stage parasitemia on the day of transfection. For transfection, cultures were pelleted, and pellets were washed with cold incomplete cytomix, then resuspended in complete cytomix (containing either 50 µg pDC2-dis-AQP2 and 400 µL transferred into an electroporation cuvette. Parasites were electroporated in a BioRad GenePulser at 310 V and 950 µF, with time constant in the range 7 to 12 ms. Parasites were washed by transfer into 10 mL chilled culture medium and pelleted; the supernatant containing debris and cytomix was removed. Transformed parasites were transferred into a 10-mL culture (3% hematocrit) and left to recover for 24 h before drug treatment. Cultures were assessed by thin smear after 2 to 3 wk; once parasitemia became detectable by thin smear, clones were isolated by limiting dilution.

### Isolation and Genotyping of Transfectant *P. falciparum* by Limiting Dilution.

Isolation of individual transfectant clones was performed by adaptation of a limited dilution protocol. Once parasitemia of transfectant cultures exceeded 4%, thin blood smears were taken to accurately count parasitemia. RBCs in the culture were enumerated in a hemocytometer to calculate the number of infected red blood cells/mL. Once the concentration of parasites in the culture was known, a tube containing exactly 10^6^ parasites/mL in complete culture medium was established, from which serial dilutions were made to prepare 10^4^ parasites/mL in complete culture medium at 1% hematocrit (HTC). From this culture, two successive 10X dilutions followed by six successive 2X dilutions into complete medium, 1% HTC, were performed, generating parasite concentrations of 1000, 100, 50, 25, 12.5, 6.25, 3.125, and 1.5625 parasites/mL. A 96-well plate was prepared, and 100 µL of each dilution was pipetted into each well of successive rows; this resulted in 100, 10, 5, 2.5, 1.25, 0.625, 0.3125, and 0.15625 parasites per well of each row A-H, respectively. The 96-well plate was maintained inside a microisolator containing malaria gas mixture. Media were changed three times per week, and HTC increased from 1 to 5% by introducing 1 µL fresh RBCs on days 4, 10, 16, and 19 after establishing the plate. Rows A-B were used to establish success of parasite growth on day 10 postsmear. If parasites were detectable by thick blood smear in control wells A/B, then on day 21 clonal rows F, G, and H were examined by thick blood smear to detect parasites. Rows F-H should all contain <1 parasite per well; rows with <35% positive wells were therefore considered successful, and clones were taken into static culture flasks to recover. Once clones were at sufficiently high parasitemia, a subset of the culture was taken for gDNA extraction followed by genotyping by PCR (with remaining parasites maintained in culture pending success of genotyping). Genotyping PCR reactions were performed on 10 ng of isolated parasite DNA using CloneAmp HiFi polymerase premix (Takara) in an Applied Biosystems thermocycler according to the manufacturer’s instructions.

### *P. falciparum* Oocyst Enumeration.

*A. coluzzii* mosquitoes infected with *P. falciparum* were killed on the date of dissection with CO_2_, followed by immersion in 70% ethanol. Carcasses were dissected in PBS under dissecting microscopes, unless at timepoints exploring sporozoite development, in which samples were dissected in 5% sodium azide. Following dissection, mosquito midguts were stained with mercurochrome for oocyst enumeration. Briefly, midguts were immersed in 0.1% mercurochrome for 20 min, prior to fixing in 4% formaldehyde for 30 min. Guts were washed in PBS and mounted in glycerol for analysis by light microscopy.

### Generation of *P. berghei* Δpbaqp2 and pbaqp2::3xha.

We used the *Plasmo*GEM vectors PbGEM_321755 and PbGEM-066039 (47) for PbAQP2 disruption and C-terminal 3xHA tagging, respectively. These vectors contain the hDHFR selection cassette that confers resistance to pyrimethamine and the negative selection cassette yFCU that substitutes delivery of 5-fluorocytosine (48). The targeting cassettes were released by NotI digestion resulting in 74% deletion of PbAQP2 coding sequence at the gene 5′ end. Transfection, drug selection of transgenic parasites, and clonal selection by dilution cloning were carried out in the *c507* line as described (20).

### Parasite Genotypic Analysis.

*P. berghei* gDNA was extracted from blood sampled from positive mice using the DNeasy kit (QIAGEN) following the manufacturer’s instructions. *P. falciparum* parasite cultures at 8 to 10% parasitemia were pelleted and washed with cold PBS. Cells were resuspended in cold saponin (0.05% in PBS) lysis buffer and left on ice for 5 min. Lysate was centrifuged (6,000 rcf, 5 min), and gDNA was extracted from pellets also using the DNeasy kit (QIAGEN). Successful gene modification events or maintenance of the wild-type loci were performed by PCR using primers listed in *SI Appendix*, Table S5.

### Exflagellation Assays.

Blood from *P. berghei*–infected mice exhibiting 8 to 10% gametocytemia or from *P. falciparum* gametocyte cultures was added to RPMI medium (RPMI 1640, 20% v/v FBS, and 100 μM xanthurenic acid, pH 7.4) in a 1:40 ratio and incubated for 10 min. Exflagellation events were counted in a standard hemocytometer under a light microscope.

### *P. berghei* Macrogamete to Ookinete Conversion Assays.

Blood from mice exhibiting 8 to 10% gametocytemia was added to RPMI medium (RPMI 1640, 20% v/v FBS, and 100 μM xanthurenic acid, pH 7.4) and incubated for 24 h at 21 °C to allow for ookinete formation. This suspension was then incubated with a Cy3-labeled 13.1 mouse monoclonal α-P28 (1:50 dilution) for 20 min on ice. The conversion rate was calculated as the percentage of Cy3-positive ookinetes to Cy3-positive macrogametes and ookinetes.

### *P. berghei* Mosquito Infections and Quantifications.

*A. coluzzii* Ngousso mosquitoes were infected by direct feeding on mice infected with *P. berghei* at a parasitemia and gametocytemia of 5 to 6% and 1 to 2%, respectively. To determine oocyst load, midguts were dissected at 7 to 10 d pbf and fixed in 4% paraformaldehyde (PFA). To determine the sporozoite load, 25 to 30 midguts and salivary glands were dissected at 15 and 21 d pbf, respectively. The dissected tissues were washed in PBS and subsequently homogenized. Sporozoites were counted in a standard hemocytometer under a light microscope. To assess mosquito-to-mouse transmission, about 30 *A. coluzzii* mosquitoes that had blood-fed on *P. berghei*–infected mice 21 d earlier were allowed to feed on anesthetized C57/BL6 mice. Mouse parasitemia was monitored daily until 14 d post mosquito bite by Giemsa staining.

### Western Blot Analysis.

For extraction of cell lysate for analysis, purified blood stages, gametocytes and ookinetes were suspended in whole cell lysis buffer (1XPBS, 1% v/v Triton X-100). Proteins resolved by SDS-PAGE were transferred to a polyvinylidene difluoride membrane. Detection was performed using rabbit α-HA (Cell Signaling Technology) (1:1,000), goat α-GFP (Rockland Chemicals) (1:1,000), and 13.1 mouse monoclonal α-P28 (1:1,000) antibodies. Secondary horseradish peroxidase-conjugated goat α-rabbit IgG, goat α-mouse IgG antibodies (Promega), and donkey α-goat IgG (Abcam) were used at 1: 2,500, 1: 2,500 and 1: 5,000 dilutions, respectively. All primary and secondary antibodies were diluted in 5% w/v milk-PBS-Tween (0.05% v/v) blocking buffer.

### Indirect Immunofluorescence and Live Fluorescent Assays.

*P. berghei* blood stage gametocytes, ookinetes, and sporozoites were fixed in 4% PFA in PBS for 10 min at room temperature. Fixed parasites were washed 3X with 1XPBS for 10 min each and then smeared on glass slides. Permeabilization of the parasites was done using 0.2% v/v Triton X-100 in PBS for 10 min at room temperature. Permeabilized parasites were washed 3 times in PBS for 10 min each and blocked with 1% w/v bovine serum albumin in PBS for 1 h at room temperature. Parasites were stained with rabbit α-HA (CST) (1:1,000), rabbit α-GFP (ThermoFisher) (1:500), 13.1 mouse monoclonal α-P28 (Ref) (1:1,000), and 2A10 mouse monoclonal α-PfCSP (1:100) antibodies. Alexa Fluor rabbit 488 and mouse 568 conjugated secondary goat antibodies (ThermoFisher) were used at a dilution of 1:1,000. DAPI was used to stain nuclear DNA. Images were acquired using a Leica SP5 MP confocal laser-scanning microscope and visualized using Image J.

For live fluorescent assays, purified live 24 h mature ookinetes were co-stained with LysoTracker™ Red DND-99 (ThermoFisher) and Hoechst 33342 (ThermoFisher). LysoTracker Red and Hoechst were diluted in PBS and utilized at a final concentration of 75 nM and 1 mg/mL, respectively. The diluted staining solution was preincubated at 37 °C. The was then added to the purified ookinetes and incubated at 21 °C for 20 to 30 min. The ookinete preparation was then loaded onto a microscope slide, and a coverslip was applied for fluorescence observations.

### Electron Microscopy.

Infected midguts were dissected in PBS at 24 h pbf, fixed with 4% PFA, postfixed in 1% osmium tetroxide, rinsed, dehydrated through a graded ethanol series, and embedded in Epon-Araldite resin. Following screening of semifield sections, ultrathin sections were transferred to copper grids and poststained with uranyl acetate and lead citrate. Sections were observed at 80 kV on a Philips Biotwin CM120 transmission electron microscope.

### Statistical Analysis.

All statistical analyses were performed using GraphPad Prism v8.0.

## Supplementary Material

Appendix 01 (PDF)

## Data Availability

All study data are included in the article and/or *SI Appendix*.
