# Supplementary material for: Intracellular Plasmodium aquaporin 2 is important for sporozoite production in the mosquito vector and malaria transmission
Source: Proc Natl Acad Sci U S A. 2023 Oct 26;120(44):e2304339120. doi: 10.1073/pnas.2304339120 (PMC10622946; doi:10.1073/pnas.2304339120)
Supplement: Supplementary file 1 — Appendix 01 (PDF) [file pnas.2304339120.sapp.pdf]

PNAS

Supporting Information for

Intracellular *Plasmodium* aquaporin 2 is important for sporozoite production in the mosquito vector and malaria transmission

Alexander J. Bailey, Chiamaka Valerie Ukegbu, Maria Giorgalli, Tanguy Rene Balthazar Besson, George K. Christophides, Dina Vlachou

George K. Christophides or Dina Vlachou

Email: d.vlachou@imperial.ac.uk or g.christophides@imperial.ac.uk

This PDF file includes:

Figures S1 to S9

Tables S1 to S5

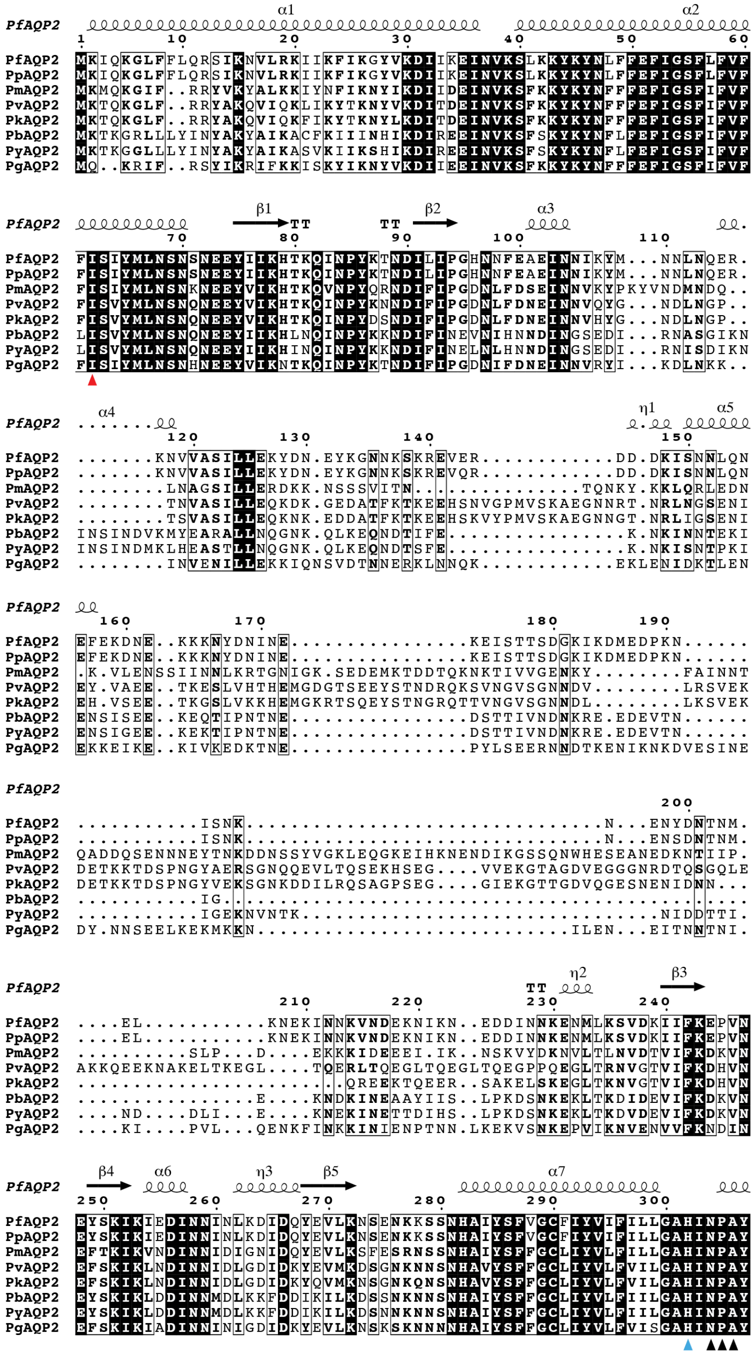

PfAQP2 α1 α2
PfAQP2 MKIQKGLFFLQRSIKNVLRKIIKFIKGYVKDIIKEINVKSLKKYKYNLFFEFIGSFLFVF
PpAQP2 MKIQKGLFFLQRSIKNVLRKIIKFIKGYVKDIIKEINVKSLKKYKYNLFFEFIGSFLFVF
PmAQP2 MKMQKGIF..RRYVKYALKKIYNFIKNYIKDIIDEINVKSFKKYKYNFFFEFIGSFIFVF
PvAQP2 MKTQKGLF..RRYAKQVIQKLIKYTKNYVKDITDEINVKSFKKYKYNFFFEFIGSFIFVF
PkAQP2 MKTQKGLF..RRYAKQVIQKFIKYTKNYLKDITDEINVKSFKKYKYNFFFEFIGSFIFVF
PbAQP2 MKTKGRLLLYINYAKYAIKACFKIIINHIKDIREEINVKSFSKYKYNFLFEFIGSFIFVF
PyAQP2 MKTKGGLLLYINYAKYAIKASVKIIKSHIKDIREEINVKSFSKYKYNFLFEFIGSFIFVF
PgAQP2 MQ..KRIF..RSYIKRIFKKISKYIKNYVKDIIEEINVKSFKKYKYNFFFEFIGSFIFVF
PfAQP2 β1 TT TT β2 α3
PfAQP2 FISIYMLNSNSNEEYIIKHTKQINPYKTNDILIPGHNNFEAEINNIKYM...NNLNQER.
PpAQP2 FISIYMLNSNSNEEYIIKHTKQINPYKTNDILIPGHNNFEAEINNIKYM...NNLNQER.
PmAQP2 FISIYMLNSNKNEEYVIKHTKQVNPYQRNDIFIPGDNLFDSEINNVKYPKYVNDMNDQ..
PvAQP2 FISVYMLNSNQNEEYVIKHTKQINPYKNNDIFIPGDNLFDNEINNVQYG...NDLNGP..
PkAQP2 FISVYMLNSNQNEEYVIKHTKQINPYDSNDIFIPGDNLFDNEINNVHYG...NDLNGP..
PbAQP2 LISVYMLNSNQNEEYIIKHLNQINPYKKNDIFINEVNIHNNDINGSEDI...RNASGIKN
PyAQP2 LISVYMLNSNQNEEYIIKHINQINPYKKNDIFINELNLHNNDINGSEDI...RNISDIKN
PgAQP2 FISIYMLNSNHNEEYVIKNTKQINPYKTNDIFIPGDNIFDNEINNVRYI...KDLNKK..
α4 η1 α5
PfAQP2
PfAQP2 .......KNVVASILLEKYDN.EYKGNNKSKREVER...........DD.DKISNNLQN
PpAQP2 .......KNVVASILLEKYDN.EYKGNNKSKREVQR...........DD.DKISNNLQN
PmAQP2 ........LNAGSILLERDKK.NSSSVITN...............TQNKY.KKLQRLEDN
PvAQP2 ........TNVASILLEQKDK.GEDATFKTKEEHSNVGPMVSKAEGNNRT.NRLNGSENI
PkAQP2 ........TSVASILLEQKNK.EDDATFKTKEEHSKVYPMVSKAEGNNGT.NRLIGSENI
PbAQP2 INSINDVKMYEARALLNQGNK.QLKEQNDTIFE................K.NKINNTEKI
PyAQP2 INSINDMKLHEASTLLNQGNK.QLKEQNDTSFE................K.NKISNTPKI
PgAQP2 ........INVENILLEKKIQNSVDTNNERKLNNQK...........EKLENIDKTLEN
PfAQP2
PfAQP2 EFEKDNE..KKKNYDNINE...............KEISTTSDGKIKDMEDPKN......
PpAQP2 EFEKDNE..KKKNYDNINE...............KEISTTSDGKIKDMEDPKN......
PmAQP2 .K.VLENSSIINNLKRTGNIGK.SEDEMKTDDTQKNKTIVVGENKY........FAINNT
PvAQP2 EY.VAEE..TKESLVHTHEMGDGTSEEYSTNDRQKSVNGVSGNNDV........LRSVEK
PkAQP2 EH.VSEE..TKGSLVKKHEMGKRTSQEYSTNGRQTTVNGVSGNNDL........LKSVEK
PbAQP2 ENSISEE..KEQTIPNTNE...............DSTTIVNDNKRE.EDEVTN......
PyAQP2 ENSIGEE..KEKTIPNTNE...............DSTTIVNDNKRE.EDEVTN......
PgAQP2 EKKEIKE..KIVKEDKTNE...............PYLSEERNNDTKENIKNKDVESINE
PfAQP2
PfAQP2 ...........ISNK................................N...ENYDNTNM.
PpAQP2 ...........ISNK................................N...ENSDNTNM.
PmAQP2 QADDQSENNNEYTNKDDNSSYVGKLEQGKEIHKNENDIKGSSQNWHESEANEDKNTIIP.
PvAQP2 DETKKTDSPNGYAERSGNQQEVLTQSEKHSEG....VVEKGTAGDVEGGGNRDTQSGQLE
PkAQP2 DETKKTDSPNGYVEKSGNKDDILRQSAGPSEG....GIEKGTTGDVQGESNENIDNN...
PbAQP2 ....IG...
PyAQP2 ...........IGEKNVNTK............................NIDDTTI.
PgAQP2 DY.NNSEELKEKMKKN.............................ILEN...EITNNTNI.
PfAQP2 TT η2 β3
PfAQP2 ....EL...........KNEKINNKVNDEKNIKN..EDDINNKENMLKSVDKIIFKEPVN
PpAQP2 ....EL...........KNEKINNKVNDEKNIKN..EDDINNKENMLKSVDKIIFKEPVN
PmAQP2 ..........SLP...D....EKKKIDEEEI.IK..NSKVYDKNVLTLNVDTVIFKDKVN
PvAQP2 AKKQEEKNAKELTKEGL....TQERLTQEGLTQEGLTQEGPPQEGLTRNVGTVIFKDHVN
PkAQP2 ....QREEKTQEER..SAKELSKEGLTKNVGTVIFKDHVN
PbAQP2 .................E....KNDKINEAAYIIS..LPKDSNKEKLTKDIDEVIFKDKVN
PyAQP2 ....ND....DLI....E....KNEKINETTDIHS..LPKDSNKEKLTKDVDEVIFKDKVN
PgAQP2 ....KI....PVL....QENKFINKKINIENPTNN.LKEKVSNKEPVIKNVENVVFKNDIN
β4 α6 η3 β5 α7
PfAQP2
PfAQP2 EYSKIKIEDINNINLKDIDQYEVLKNSENKKSSNHAIYSFVGCFIYVIFILLGAHINPAY
PpAQP2 EYSKIKIEDINNINLKDIDQYEVLKNSENKKSSNHAIYSFVGCFIYVIFILLGAHINPAY
PmAQP2 EFTKIKVNDINNIDIGNIDQYEVLKSFESRNSSNHAIYSFFGCLIYVLFILLGAHINPAY
PvAQP2 EFSKIKLNDINNIDLGDIDKYEVMKDSGNKNNSNHAVYSFFGCLIYVLFVILGAHINPAY
PkAQP2 EFSKIKLNDINNIDLGDIDKYQVMKNSGNKQNSNHAVYSFFGCLIYVLFVILGAHINPAY
PbAQP2 EYSKIKLDDINNMDLKKFDDIKILKDSSNKNNSNHAIYSFFGCLIYVLFIILGAHINPAY
PyAQP2 EYSKIKLDDINNMDLKKFDDIKILKDSNNKNNSNHAIYSFFGCLIYVLFIILGAHINPAY
PgAQP2 EFSKIKIADINNINIGDIDKYEVLKNSKSKNNNNHAIYSFFGCLIYVLFVISGAHINPAY

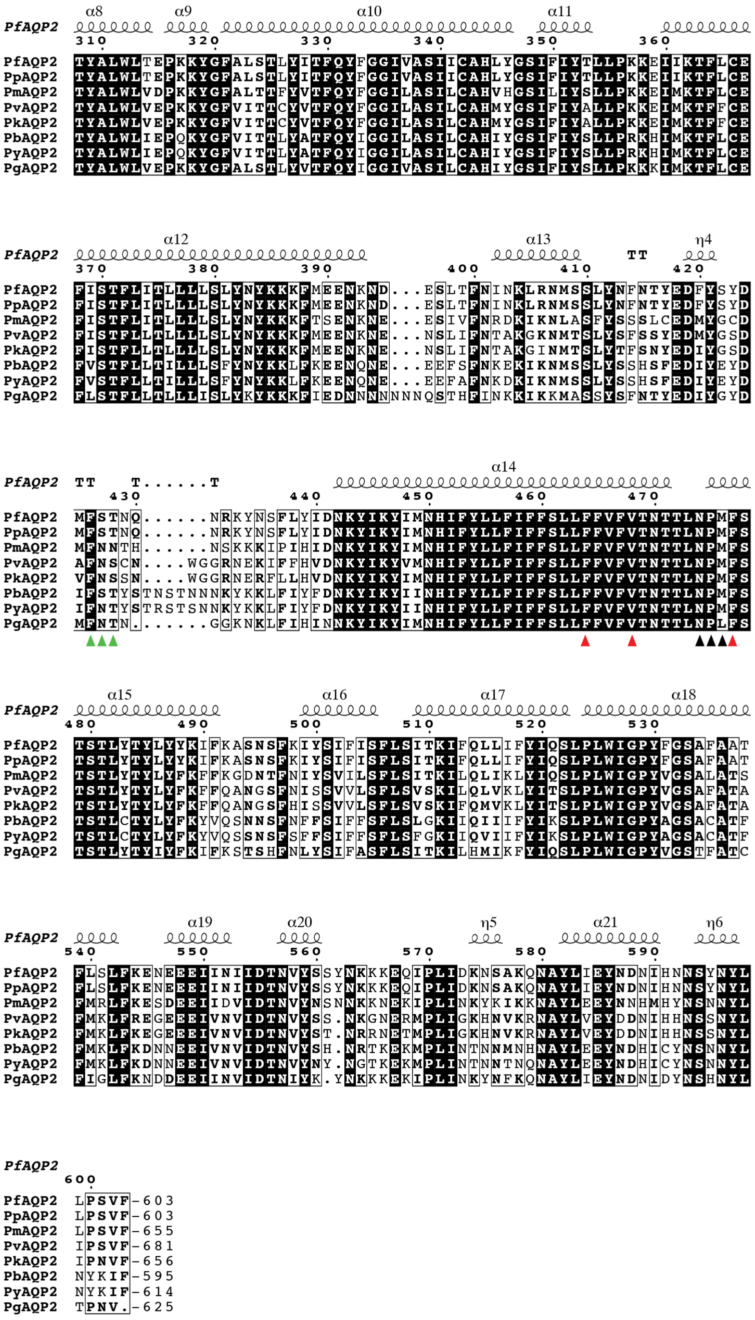

Fig. S1. Multiple sequence alignment of *Plasmodium* AQP2 orthologs. AQP2s from eight *Plasmodium* species are included: *P. falciparum* (PfAQP2, PF3D7_0810400), *P. praefalciparum* (PpAQP2, PPRFG01_0811800), *P. malariae* (PmAQP2, PmUG01_14045900), *P. vivax* (PvAQP2, PVP01_1429900), P. *knowlesi* (PkAQP2, PKNH_1430300), *P. berghei* (PbAQP2, PBANKA_1427100), *P. yoelli* (PyAQP2, PY17X_1429200) and *P. gallinaceum* (PgAQP2, PGAL8A_00525700). Multiple sequence alignment is performed with t-coffee, while secondary structure predictions of PfAQP2 were obtained from Alphafold Monomer v2.0 predictions, annotated using ESPript3.0 and presented above the alignment. The selectivity filter residues are highlighted with red triangles and the histidine residue participating in the pore with a cyan triangle.

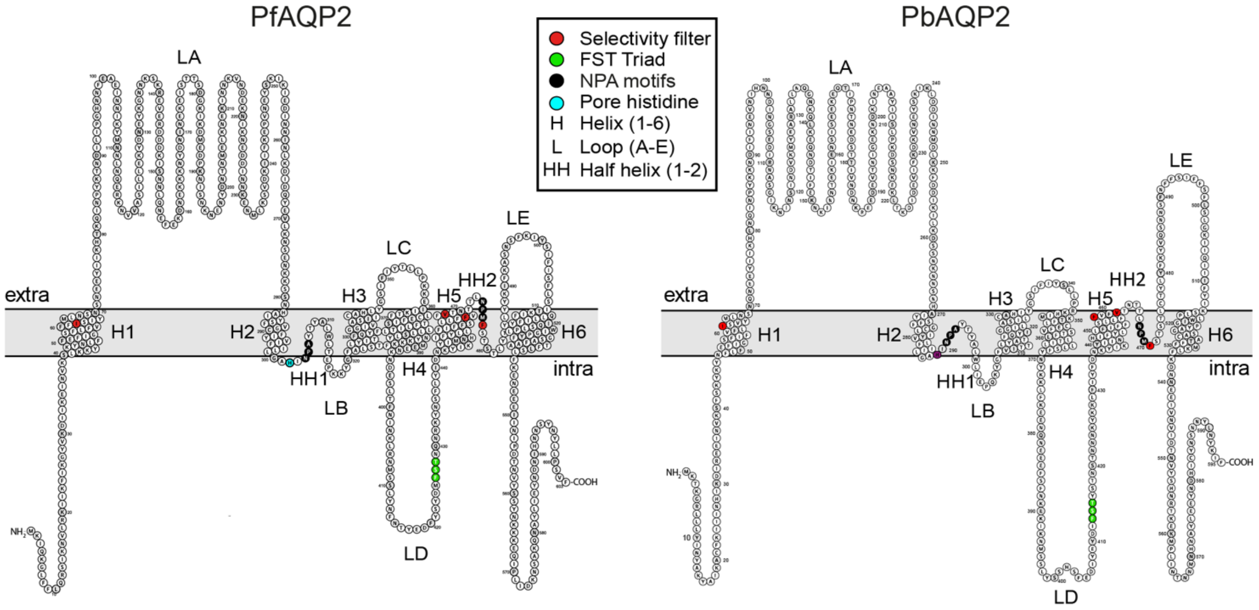

Fig. S2. Predicted membrane topology of PfAQP2 (left) and PbAQP2 (right). Topology of is predicted with Alphafold and PPM, and visualized using Protter. Transmembrane helices are labeled as H1-6, half-helices as HH1-2, and extramembrane loops as LA-E. Key residues are highlighted: selectivity filter in red, NPA/NPM motifs in black, FST triad of loop D in green, and histidine participating in the pore in cyan.

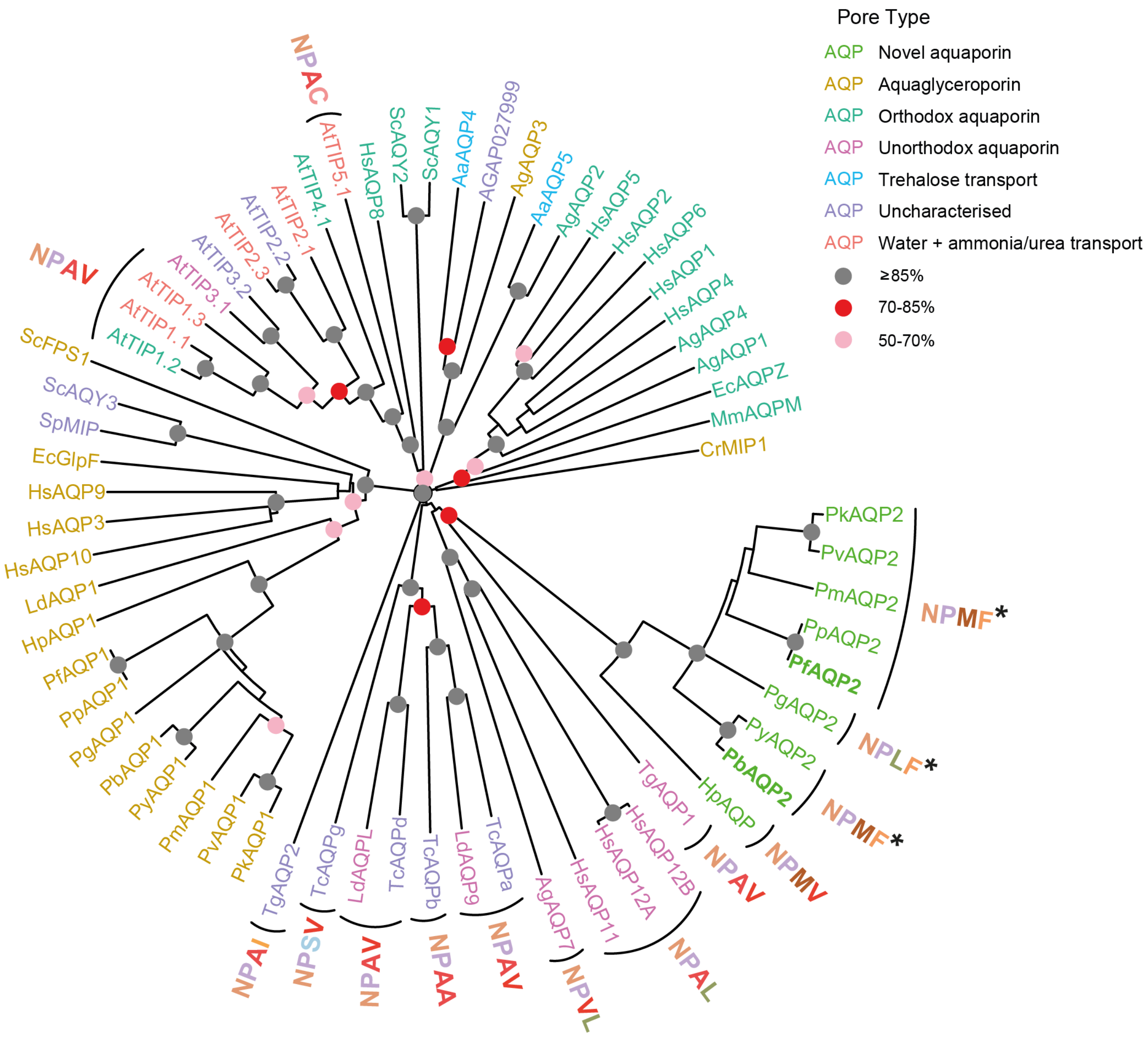

Fig. S3. Phylogenetic tree of the conserved aquaporin pore-forming regions. 66 aquaporin genes from 21 species are presented: *P. falciparum* (Pf), *P. berghei* (Pb), *P. praefalciparum* (Pp), *P. malariae* (Pm), *P. vivax* (Pv), *P. knowlesi* (Pk), *P. yoelli* (Py), *P. gallinaceum* (Pg), *Hepatocystis sp.* (Hp), *Toxoplasma gondii* (Tg), *Trypanosoma cruzii* (Tc), *Leishmania donovani* (Ld), *Homo sapiens* (Hs), *Anopheles gambiae* (Ag), *Aedes aegypti* (Aa), *Arabidopsis thaliana* (At), *Saccharomyces cerevisiae* (Sc), *Schizosaccharomyces pombe* (Sp), *Escherichia coli* (Ec), *Methanothermobacter marburgensis* (Mm), *Chlamydomonas reinhardtii* (Cr). Ar/R selectivity filter motifs with arginine substitutions are annotated (canonical sequence: NPAR), colored by amino acid sequence. The *Plasmodium* AQP2 genes with unique phenylalanine substitution are highlighted with an asterisk. Gene names are coloured by category of pore type and nodes are labeled with bootstrap confidence values derived from 1000 iterations as indicated in the key.

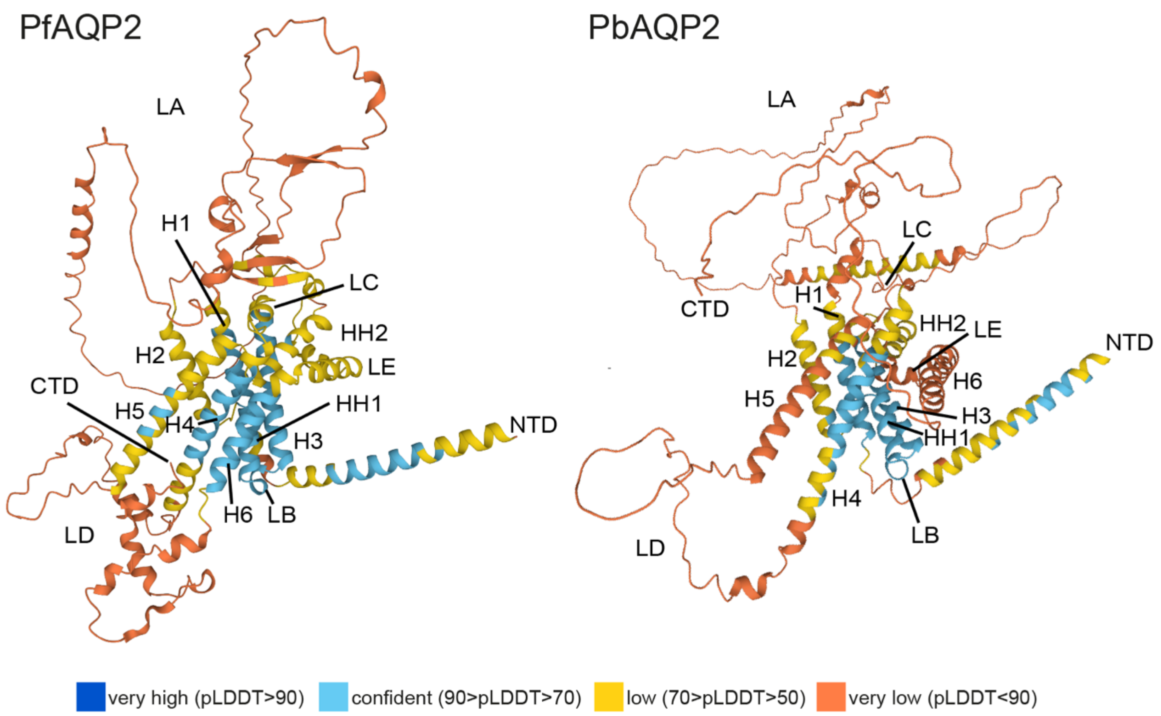

Fig. S4. Putative 3D structures of PfAQP2 and PbAQP2. Protein structures are predicted by Alphafold Monomer v2.0 and can be accessed in UniProt, PlasmoDB and other databases as C0H4T7 and A0A509AUY5, respectively. Structures are colored by Alphafold prediction confidence values as shown in the key below the structures.

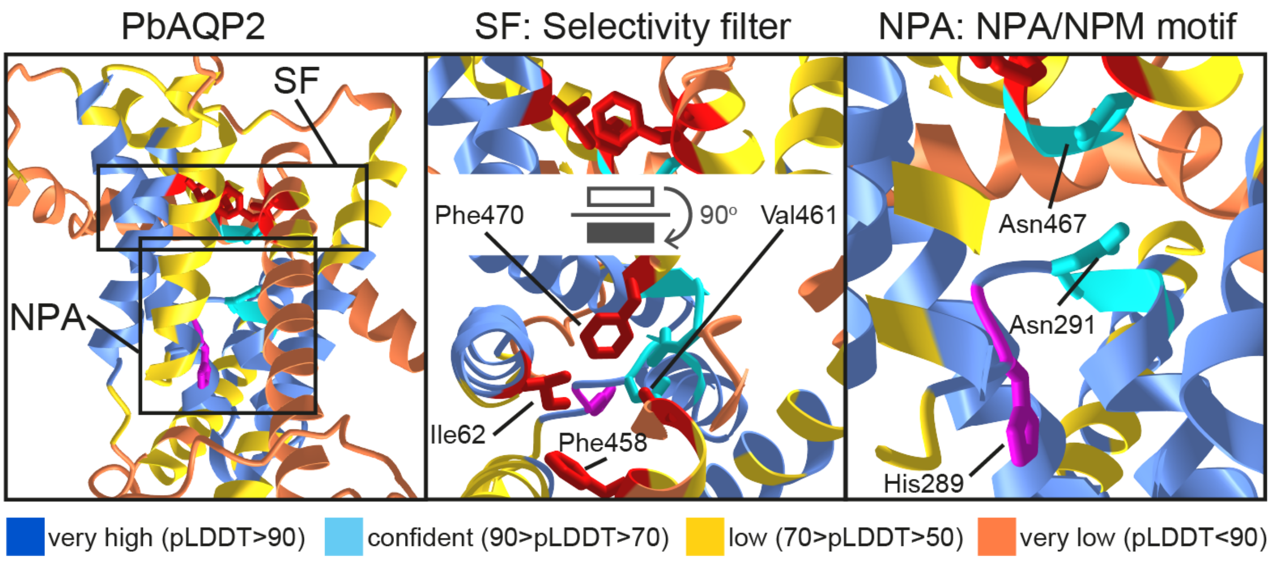

Fig. S5. Predicted structure of PbAQP2 pore and selectivity filter. Structures are predicted with Alphafold Monomer v2.0 on the CoLab server. Top ranking models are shown. Structures are colored by confidence level as shown in the key below the figure. Residues contributing to the selectivity filter are shown in red and those contributing to the NPA/NPM motif are shown in cyan, while the histidine residue participating in the pore is shown in magenta.

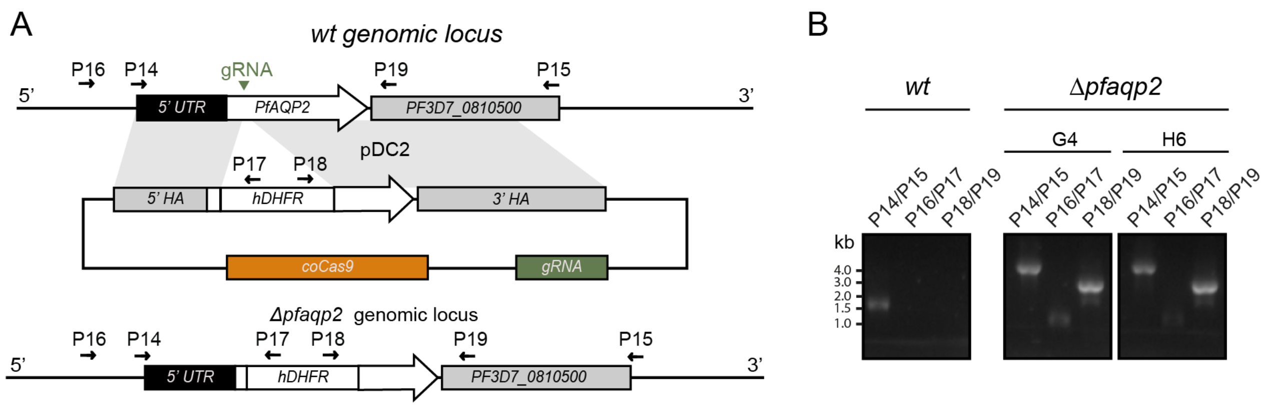

Fig. S6. Schematic diagram of *Δpfaqp2* knockout generation by CRISPR-Cas9. (*A*) The endogenous *NF54* PfAQP2 locus shown at the top was disrupted using the AQP2-dis-pDC2 plasmid construct shown in the center of the panel resulting in the disrupted locus shown at the bottom. For the generation of the plasmid construct, primers described in Table S5 are used to clone the 5′ and 3′ donor homology arms (HA) upstream and downstream of the Cas9 cut site on the parasite genome, respectively. The donor AQP2-dis-pDC2 plasmid includes the hDHFR selectable marker cassette. (*B*) PCR genotypic analysis of successfully generated recombinants (left picture) and isolated *Δpfaqp2* clonal populations (right picture) using primers specific for recombinant genomic sequence.

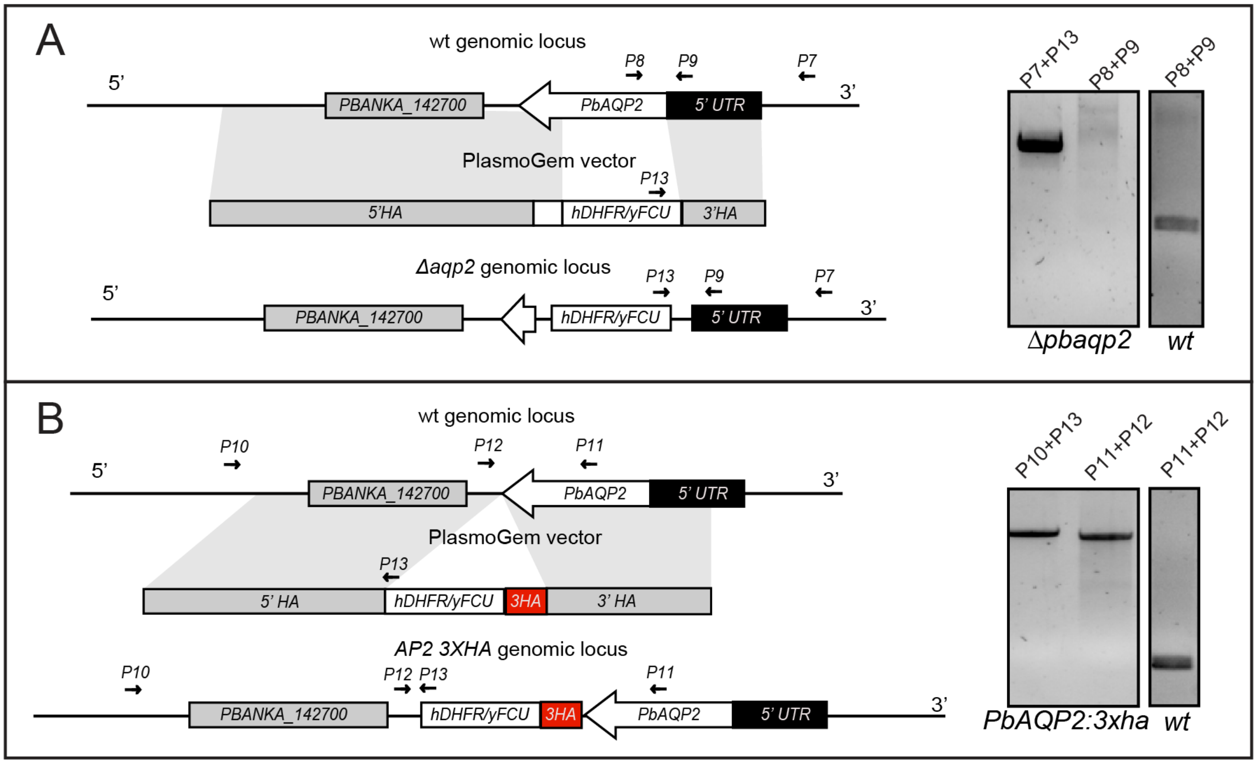

Fig. S7. Generation of *Δpbaqp2* mutant and gene tagged parasites. (*A*) Schematic representation of the disruption of *P. berghei* in the *c507* line, using *Plasmo*GEM vectors carrying the *human DHFR* (*hDHFR)/yFCU* pyrimethamine resistance cassette. In each panel, the *wt* genomic locus (top), the transfected DNA fragment carrying the homologous arms (HA) and the selectable markers (middle), and the final transgenic locus after gene disruption by double crossover homologous recombination (bottom) are shown. Constructs are not drawn to scale. Small arrows show the primers used for the confirmation of integration of gene targeting constructs and deletion of the gene of interest using PCR as shown in the panels on the right. The *c507 wt* parental parasites were used as a control for detection of the *wt* locus. (*B*) Schematic representation of the 3XHA tagging of in the c507 line by using PlasmoGEM gene tagging vectors that carry the hDHFR)/yFCU selectable marker. In each panel, the wt genomic locus (top), the plasmid carrying the homologous arms (HA), the transfected DNA fragment carrying the tagging cassettes and the selectable markers (middle), and the final transgenic locus after double crossover homologous recombination (bottom) are shown. Constructs are not drawn to scale. Small arrows show the primers used for the confirmation of integration of gene targeting constructs and deletion of the gene of interest using diagnostic PCR reactions as shown in the panels on the right.

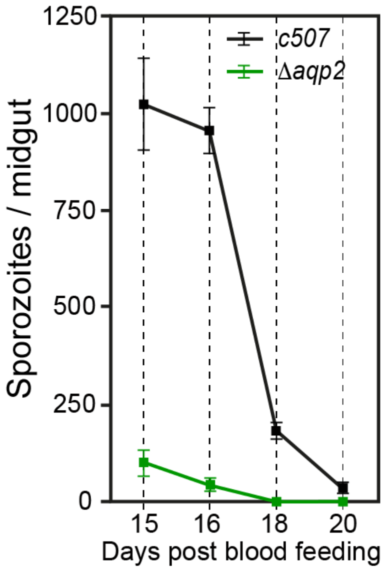

Fig. S8. Sporozoite production in *Δpbaqp2* mutant oocysts. Midgut sporozoite load in *Δpbaqp2* and *c507* (control) infected *A. coluzzii* mosquitoes at 15, 16, 18 and 20 days pbf. The mean of the pooled data from two biological replicates is shown. The number of sporozoites was from suspensions of 20 homogenized midguts, assayed in two batches of ten. Error bars show standard error.

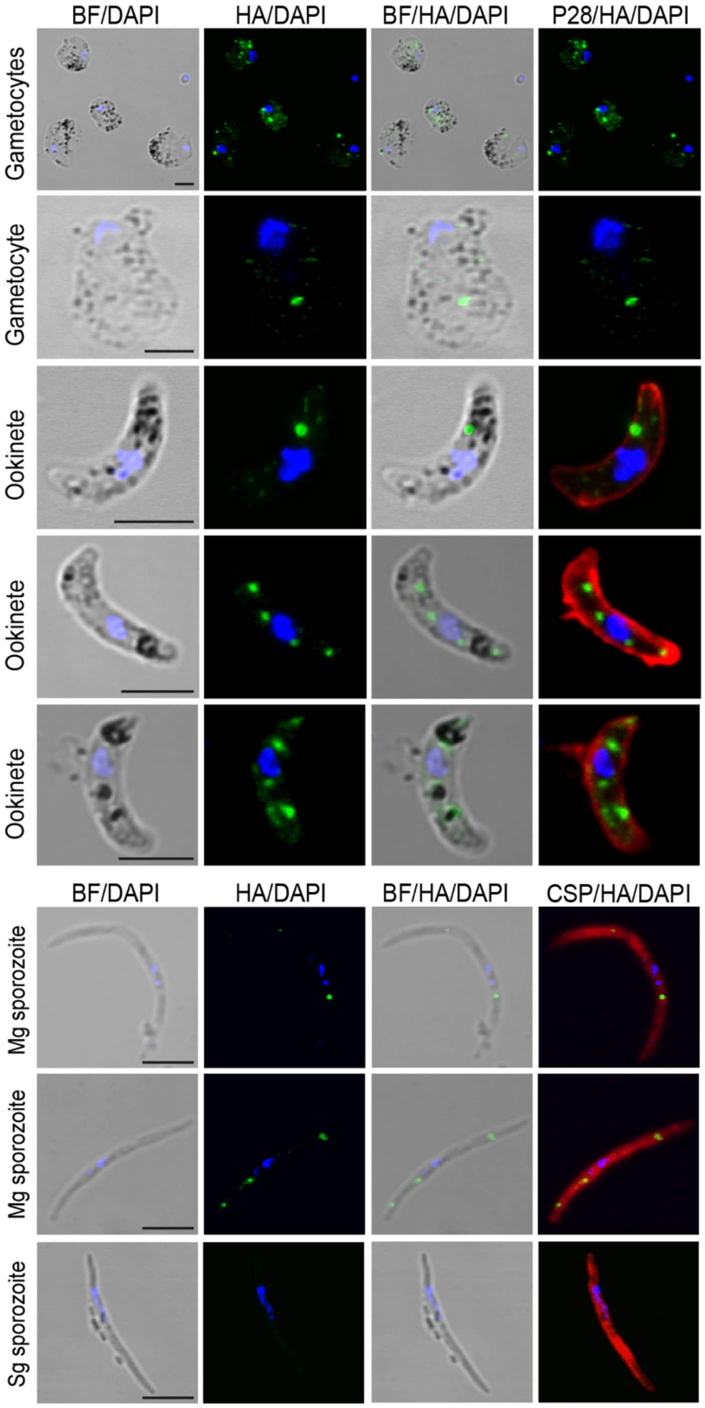

Fig. S9. Subcellular localization of *P. berghei* AQP2. Immunofluorescence assays of *aqp2::3xha* purified gametocytes, *in vitro* cultured ookinetes sampled 24 hours after culture setup, and sporozoites obtained from infected *A. coluzzii* midguts 9 days pbf. AQP2::3xHA (green) and P28 (red, ookinetes) or CSP (red, sporozoites) are detected using antibodies. DNA is stained with DAPI (blue). BF is brightfield. Scale bar is 2.5 μM. Mg: Midgut, Sg: Salivary gland.

Table S1. Oocyst counts of control *Δpfaqp2* mutant parasites and NF54 controls in *A. coluzzii*

Replicate	Parasite	No of midguts	Prevalence (%)	Arithmetic mean	Median	Parasite Range	*P* value
Pool	NF54	139	69.8	2.25	1	28	
	G4	110	65.5	1.74	1	15	0.7988
	H6	141	68.1	1.82	1	24	0.8858
R1	NF54	22	54.5	0.77	1	3	
	G4	22	50.0	0.86	0.5	5	>0.9999
	H6	25	72.0	1.44	1	6	0.2312
R2	NF54	21	57.1	1.91	1	12	
	G4	18	55.6	0.78	1	3	0.6934
	H6	27	55.6	0.96	1	3	>0.9999
R3	NF54	61	75.4	2.95	2	28	
	G4	30	73.3	2.23	2	7	>0.9999
	H6	44	70.5	2.39	1.5	24	0.6039
R4	NF54	35	77.1	2.17	1	11	
	G4	40	72.5	2.28	2	15	>0.9999
	H6	45	71.1	2.00	2	10	>0.9999

Infection data from four biological replicates of *A. coluzzii* infected with *Δpfaqp2* (clones G4 and H6) *and NF54* parasite lines, assessed at 9 days post-infection. *P* values were calculated using the Kruskal-Wallis test, comparing to wild type, and adjusted with Dunn's multiple comparisons test.

Table S2. Sporozoite numbers of control *NF54* and *Δpfaqp2* parasites in *A. coluzzii* infections

	Midgut sporozoites			Salivary gland sporozoites		
Parasite	Mean	SEM	P value	Mean	SEM	P value
NF54	3717	967.1		1149	211.0	
G4	30.4	8.7	< 0.0001	28.5	13.5	< 0.0001
H6	30.5	16.7	< 0.0001	20	6.6	< 0.0001

Mean oocyst and salivary gland sporozoite numbers of *A. coluzzii* infected with *Δpfaqp2* (clones G4 and H6) *and NF54* parasite lines. Pooled data from 4 biological replicates are presented. In each replicate, sporozoite numbers were determined from 25-30 homogenized mosquito midguts or salivary glands at 11 and 18 days pbf, respectively. *P* values were calculated by one-way ANOVA, comparing to wild type, and adjusted with Dunnett's multiple comparisons test. SEM represents standard error of mean.

Table S3. Oocyst numbers of control *c507* and *Δpbaqp2* parasites in *A. coluzzii* infections

Replicate	Parasite	No. of midguts	Prevalence (%)	Mean	Median	Range	*P* value
Pool	c507	61	82	22.1	13	0-176	
	Δpbaqp2	64	78	16.4	9	0-185	0.2284
R1	c507	32	81	22.8	13	0-176	
	Δpbaqp2	31	84	13.1	9	0-57	0.581
R2	c507	29	83	21.2	14	0-114	
	Δpbaqp2	33	73	19.5	8	0-185	0.2941

Data are from two biological replicates of infected *A. coluzzii* dissected at 8 days pbf. *P* values were calculated using the Mann-Whitney t-test.

Table S4. Sporozoite numbers of control *c507* and *Δpbaqp2* parasites in *A. coluzzii* infections

	Midgut sporozoites			Salivary gland sporozoites		
Parasite	Mean	SEM	P value	Mean	SEM	P value
c507	3,567	259		1,843	83	
Δpbaqp2	199	58	0.0115	34	24	0.0045

Mean oocyst and salivary gland sporozoite numbers from *A. coluzzii* infections. Pooled data from two biological replicates are presented. In each replicate, sporozoite numbers were determined from 25-30 homogenized mosquito midguts or salivary glands at 15 and 21 days pbf, respectively. *P* values were calculated using the unpaired Student's t-test. SEM represents standard error of mean.

Table S5. Oligonucleotide primers used in this study

Primer name	Sequence (5′ to 3′)	Description
P1 F	TATATGGGAATTTCCTTATAGGGCCCTTTAGGTTTTATTATTTTAGGG	PfAQP2 disruption upstream target
P2 R	CGCAGAGAAATCTAGAGGTACCGAGTATTGTTACCTTTATATTCGTTATC	PfAQP2 disruption upstream target
P3 F	TTACAAGCGAATTAGCTAAGCATGCGACGATGATAAAATAAGCAAT	PfAQP2 disruption downstream target
P4 R	TTCCCCGAAAAGTGCCACCTGACGTCATATAATACTGGCAACGATACC	PfAQP2 disruption downstream target
P5 F	CTCGGTACCTCTAGATTTCTC	hDHFR cassette forward primer
P6 R	GCATGCTTAGCTAATTCGCTTGTAA	hDHFR cassette reverse primer
P7: PlasmoGEM AQP2 GT F	TTGTGTTAGCTCTCTTGCCT	Pb diagnostic primer WT and KO/*c507*
P8: PlasmoGEM AQP2 QCR1	TCAACAATGCCCTTGCCTCA	Pb diagnostic primer WT and KO/*c507*
P9: PlasmoGEM AQP2 QCR2	TCATTCTACGCTTTATTAACTCA	Pb diagnostic primer WT/*c507*
P10: PlasmoGEM AQP2HA GT F	TGGGTGCGTACAATGACTAGA	Diagnostic primer tag
P11: PlasmoGEM AQP2HA QCR1	TCTGCCTCTATGGATTGGGCCA	Diagnostic primer tag
P12: PlasmoGEM AQP2HA QCR2	TGACAAGAAAAAGGTGGCAT	Diagnostic primer tag
P13: PlasmoGEM GW2	CTTTGGTGACAGATACTAC	Diagnostic primer KO and tag
P14	CTTTAGGTTTTATTATTTTAGGG	Pf diagnostic primer WT and KO
P15	CATATAATACTGGCAACGATACC	Pf diagnostic primer WT and KO
P16	GAATGTACTTATATATTTGTACACC	Pf diagnostic primer KO
P17	AACATATGTTAAATATTTATTTCTC	Pf diagnostic primer KO
P18	GTGTACTTGTTAAAATGCTATATCA	Pf diagnostic primer KO
P19	GACAGTATTCGATGCTAAGAG	Pf diagnostic primer KO
P20	TATTGAAAGTAAAAGGGAAGTGGAA	PfAQP2 disruption gRNA oligo F
P21	AAACTTCCACTTCCCTTTTACTTTC	PfAQP2 disruption gRNA oligo R
P22	AGCATTTGCCGCAACTTTCC	PfAQP2 qPCR primer F
P23	GCGCTGTTTTTATCTATTAACGGT	PfAQP2 qPCR primer R
P24	GAACCACCAAAGAGACCTTAC	EIF1α qPCR primer F
P25	TCTAGCTTCTTCCAAAACTTCC	EIF1α qPCR primer R
P26	TGAGGCAAGGGCATTGTTGA	PbAQP2 qPCR primer F
P27	TGGTAACTTCATCCTCTTCCCT	PbAQP2 qPCR primer R
P28	CACACTCATAATGTTTTCCAGTCAATT	P28 qPCR primer F
P29	AATGCACAGGTACAGGAGAAACTAAAT	P28 qPCR primer R
P30	GAATTCGTTAAACAGATCAGGGATAGTA	PbCSP qPCR primer F
P31	TTATACCAGAACCACATGTTACGTTACA	PbCSP qPCR primer R
P32	CCTGTCCTTTTACCAGACAACCA	GFP qPCR primer F
P33	GGTCTCTCTTTTCGTTGGGATCT	GFP qPCR primer R

Overlap sequences for Gibson assembly and restriction sites are shown as underlined. F, forward; R, reverse; WT, wild-type; KO, knockout. All primers are listed in a 5′ to 3′ direction.
